# Bump-and-hole engineering of human polypeptide N-acetylgalactosamine transferases to dissect their protein substrates and glycosylation sites in cells

**DOI:** 10.1016/j.xpro.2022.101974

**Published:** 2023-01-11

**Authors:** Beatriz Calle, Edgar Gonzalez-Rodriguez, Keira E. Mahoney, Anna Cioce, Ganka Bineva-Todd, Omur Y. Tastan, Chloe Roustan, Helen Flynn, Stacy A. Malaker, Benjamin Schumann

**Affiliations:** 1 Department of Chemistry, Imperial College London, London W12 0BZ, UK; 2 Chemical Glycobiology Laboratory, The Francis Crick Institute, London NW1 1AT, UK; 3 Tumour-Host Interaction Laboratory, The Francis Crick Institute, London NW1 1AT, UK; 4 Department of Chemistry, Yale University, New Haven, CT 06511, USA; 5 Structural Biology Science Technology Platform, The Francis Crick Institute, London NW1 1AT, UK; 6 Proteomics Science Technology Platform, The Francis Crick Institute, London NW1 1AT, UK

**Keywords:** Cell-based Assays, Molecular/Chemical Probes, Protein Biochemistry, Proteomics, Mass Spectrometry

## Abstract

Despite the known disease relevance of glycans, the biological function and substrate specificities of individual glycosyltransferases are often ill-defined. Here, we describe a protocol to develop chemical, bioorthogonal reporters for the activity of the GalNAc-T family of glycosyltransferases using a tactic termed bump-and-hole engineering. This allows identification of the protein substrates and glycosylation sites of single GalNAc-Ts. Despite requiring transfection of cells with the engineered transferases and enzymes for biosynthesis of bioorthogonal substrates, the tactic complements methods in molecular biology.

For complete details on the use and execution of this protocol, please refer to Schumann et al. (2020)^1^, Cioce et al. (2021)^2^, and Cioce et al. (2022)^3^

## Highlights

• Generation of bump-and-hole reporter systems for GalNAc-T glycosyltransferases
• Artificial biosynthetic pathway to biosynthesize bioorthogonal substrate analogues
• Protein substrates of individual GalNAc-Ts bioorthogonally tagged in living cells
• Substrate identification and glycan site localization achieved by MS-glycoproteomics

## Abstract (editor highlights)

Publisher’s note: Undertaking any experimental protocol requires adherence to local institutional guidelines for laboratory safety and ethics.

## Graphical abstract

**Figure:**
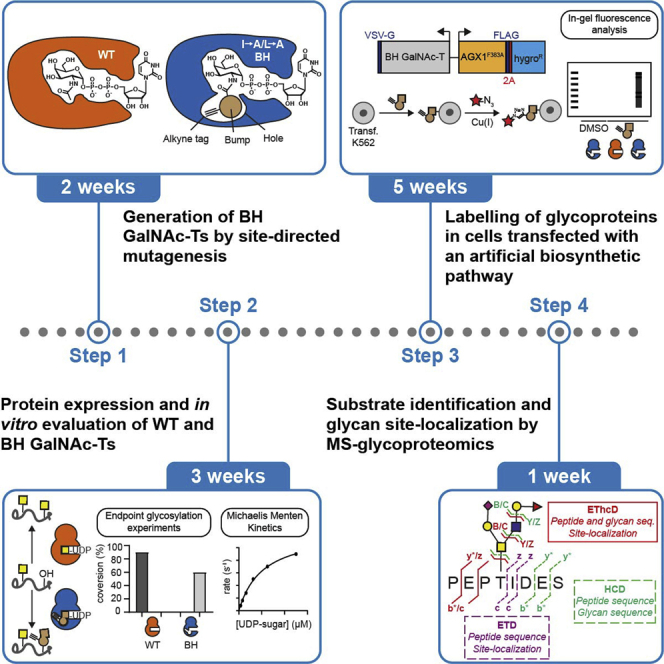

## Before you begin

**Figure 1:**
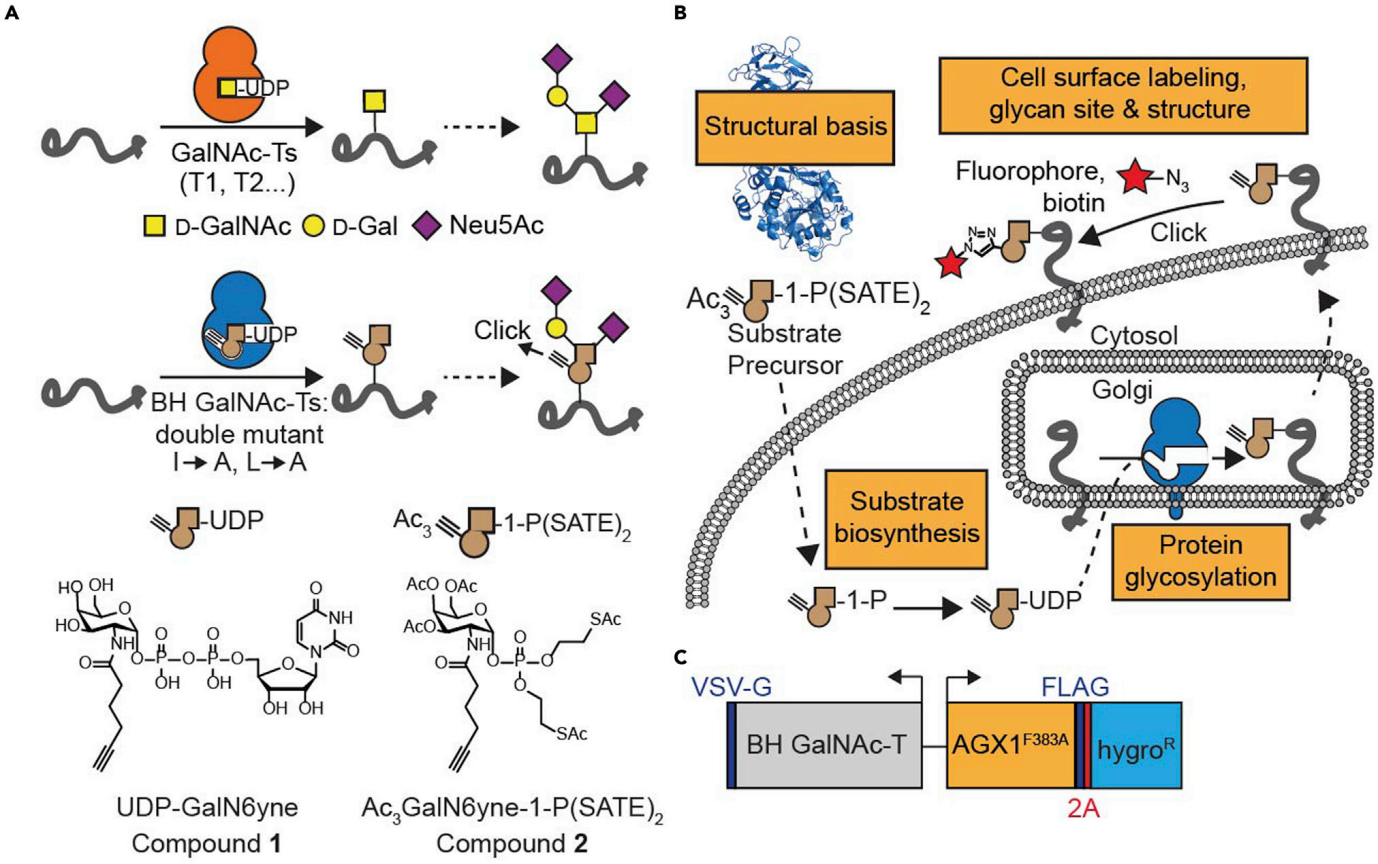
GalNAc-T bump-and-hole engineering (A) Two bulky gatekeeper residues in the active site of the BH GalNAc-T are replaced with alanines. This approach engineers the enzyme to contain a “hole” that can accept a “bumped” UDP-GalNAc analogue (UDP-GalN6yne, compound **1**). (B) Key steps required to establish a cellular GalNAc-T BH system in living cells. The cells are treated with a membrane-permeable substrate precursor (Ac_3_GalN6yne-1-P(SATE)_2_, compound **2**) which is converted to the corresponding UDP-GalNAc analogue inside the cell by an artificial biosynthetic pathway. The BH GalNAc-T then transfers the modified sugar to its protein substrates which get incorporated to the cell surface by the secretory pathway. The labelled glycoproteins can then be derivatized by click chemistry and analyzed by a variety of techniques including in-gel fluorescence and glycoproteomics. (C) BH GalNAc-T and mut-AGX1 co-expression construct used to establish the GalNAc-T BH system in cells. Figure adapted from Schumann et al.^1^ (Figure 1).

This protocol describes the creation and use of bump-and-hole-engineered (BH) *N*-acetylgalactosamine transferases 1 and 2 (GalNAc-T1 and T2) for identification of their substrate proteins and distinct glycosylation sites in the living cell. BH-GalNAc-Ts have been engineered to contain an enlarged active site (“hole”) that can accept alkyne-containing (“bumped”) substrate analogues (Figure 1A). Cells can be equipped with the ability to biosynthesize a “bumped”, chemically modified UDP-GalNAc analogue complementary to the BHGalNAc-T which allows the protein substrates of the enzyme of interest to be modified with a traceable bioorthogonal chemical tag (Figure 1B).

We detail the differential use of full-length and truncated GalNAc-T constructs. Full-length constructs—containing the transmembrane (TM), lectin, and catalytic domains—are used to establish the BH system in living cells and chemically label the cellular substrates of the GalNAc-Ts. Truncated constructs, which lack a TM domain, are used for secreted protein expression and *in vitro* glycosylation experiments.

**Figure 2:**
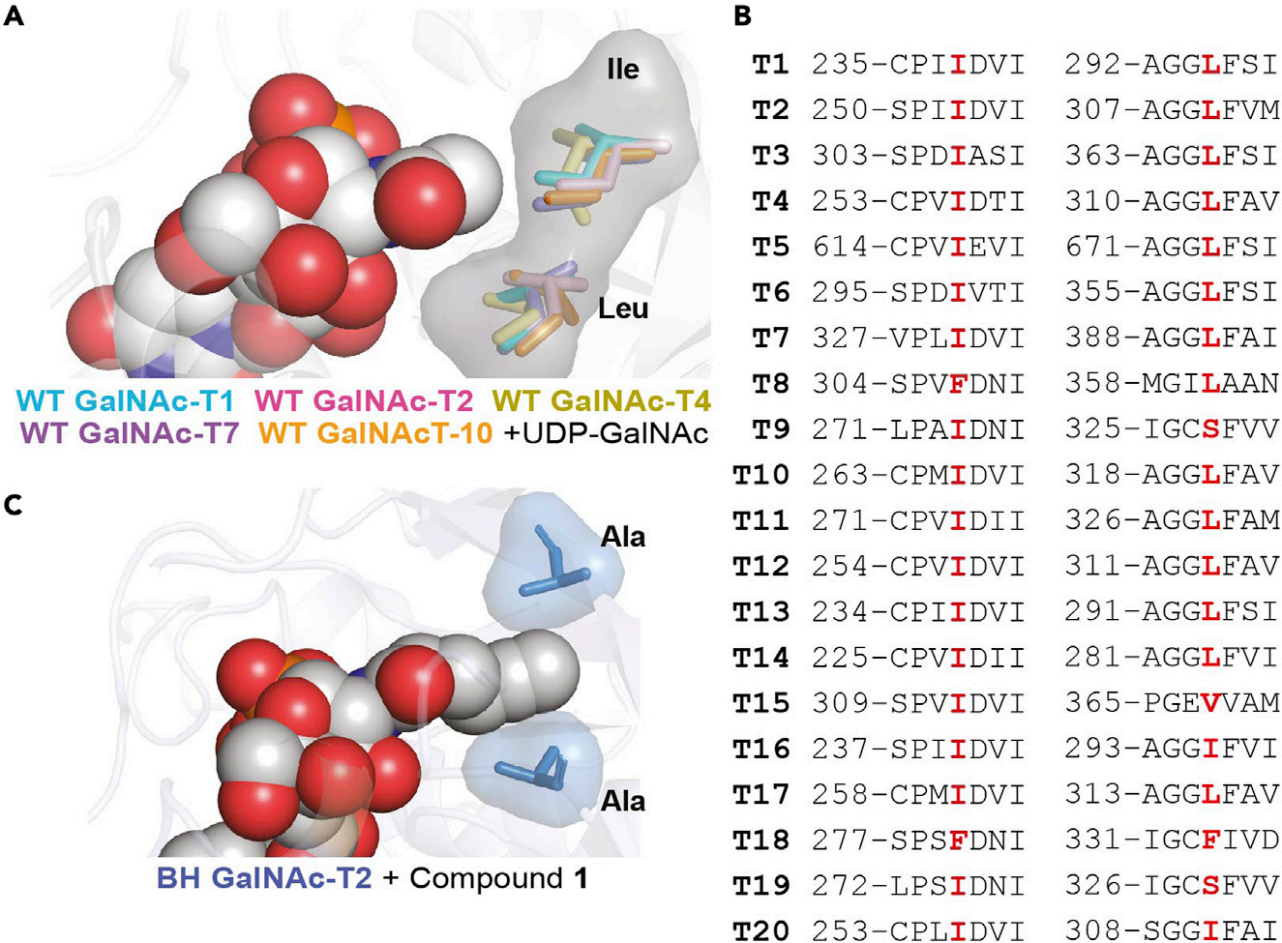
Design of gatekeeper residues (A) Gatekeeper residues identified in the crystal structures of GalNAc-T1 (PDB 1XHB), -T2 (PDB 4D0T), -T4 (PDB 5NQA), -T7 (PDB 6IWR), and -T10 (PDB 2D7I). (B) Sequence alignment of gatekeeper residues in all 20 GalNAc-Ts. (C) Co-crystal structural of BH GalNAc-T2 (PDB 6NQT) with compound **1**. Figure adapted from Cioce et al.^2^ (Figure 3).

***Note:*** This protocol was originally developed for GalNAc-T1 and GalNAc-T2 but the structural and sequence alignment observed (Figure 2) suggests potential transferability to other members of the GalNAc-T family. This protocol is optimized for its application on K-562 cells using pSBbi plasmids. We recommend using this system to first establish the viability of the protocol. Alternative cell lines or enzymes may require further optimization.

### Design of gatekeeper residues

**Timing: 1 h**

The BH system was first established using available crystal structures of GalNAc-Ts, particularly GalNAc-T1,^4^ -T2,^5^ and -T10.^6^ Recently, the human GalNAc-T3,^7^ -T4,^8^ -T7^9^ and -T12,^10^ as well as the Drosophila enzyme Pgant9,^11^ among others, have been crystallized. Bulky, hydrophobic gatekeeper residues were identified that are in close proximity to the acetamide moiety of UDP-GalNAc. Sequence and structural alignment of the 20 isoenzymes allowed mapping these gatekeeper residues across the whole enzyme family (Figures 2A and 2B). Mutation of these gatekeeper residues to alanines generated BH GalNAc-T1, T2 and T10^12^ (Figure 2C).

### Design of truncated constructs

**Timing: 30 min**

1. Inspect the sequence of the GalNAc-T to identify the location of the TM domain, the catalytic domain and the lectin domain.
  a. The sequence features in the UniProt database can be used to facilitate this process.
2. Design primers so that upon PCR amplification from cDNA sources the N-terminus of the protein is positioned immediately C-terminal to the TM domain and the catalytic and lectin domains are intact.
  a. The truncated sequence can then be cloned into an expression vector specific for the desired host system.

**Table 1:** Representative strategies for secreted protein expression of all 20 GalNAc-Ts, including the cDNA sources used in each case

GalNAc-T	Reference for secreted protein expression	cDNA source
GalNAc-T1	White et al.^13^	Human gastric cancer cell line MKN45
GalNAc-T2	White et al.^13^	Human gastric cancer cell line MKN45
GalNAc-T3	Bennett et al.^14^	Human salivary gland
GalNAc-T4	Bennett et al.^15^	Human salivary gland
GalNAc-T5	Hagen et al.^16^	Rat sublingual gland
GalNAc-T6	Bennett et al.^17^	Human salivary gland
GalNAc-T7	Bennett et al.^18^	Human gastric cancer cell line MKN45
GalNAc-T8	White et al.^19^	Human fetal brain
GalNAc-T9	Toba et al.^20^	Human brain
GalNAc-T10	Cheng et al.^21^	Human colon cancer cell line Colo205
GalNAc-T11	Schwientek et al.^22^	Human gastric cancer cell line MKN45
GalNAc-T12	Guo et al.^23^	Human lung
GalNAc-T13	Zhang et al.^24^	Human NT2RI cells
GalNAc-T14	Wang et al.^25^	Human gastric cancer cell line MKN45
GalNAc-T15	Cheng et al.^26^	Human brain
GalNAc-T16	Raman et al.^27^	Commercial sources
GalNAc-T17	Peng et al.^28^	Human small-cell lung cancer cell line Lu130
GalNAc-T18	Raman et al.^27^	Commercial sources
GalNAc-T19	Nakamura et al.^29^	Human brain
GalNAc-T20	Raman et al.^27^	Commercial sources

**Figure 3:**
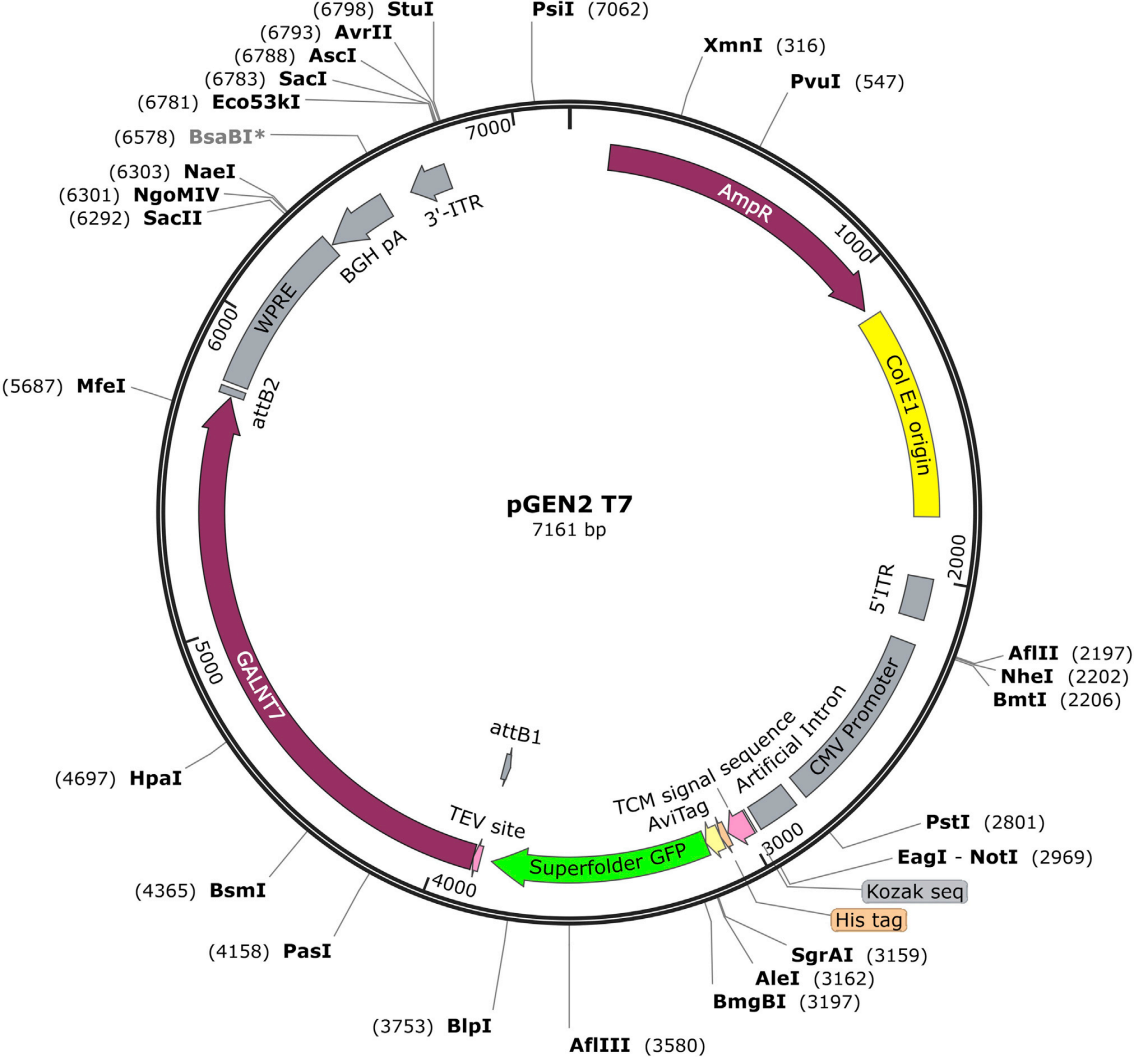
SnapGene map of the pGEN2-DEST plasmid containing GalNAc-T7 as an example, as cloned and provided by Moremen and colleagues.^30^ The BH GalNAc-T7 version of this construct was successfully expressed, purified and used in *in vitro* glycosylation experiments^41^

***Note:*** Multiple strategies have been performed to successfully express soluble forms of all 20 GalNAc-Ts (Table 1)*.* In the original publication^1^ a secretion construct of BH GalNAc-T2 was designed according to literature precedent of crystallization of the wild-type (WT) enzyme^5^ and then cloned with a His_6_ tag into a pOPING vector*.* Newer renditions have used pGEN2-DEST constructs (Figure 3) developed by Moremen et al.^30^ The provision of these vectors is an invaluable advance to the field. Vectors are commercially available in DNASU (https://dnasu.org/DNASU/Home.do) and allow secreted expression of most truncated GalNAc-Ts in mammalian cells (except GalNAc-T8, -T17, -T19 and -T20). Nevertheless, the protein expression levels produced with these constructs can vary. For this reason, alternative constructs may be required, and the expression strategies and organisms optimized.

### Cloning of BH GalNAc-Ts

**Timing: 30 min**

**Figure 4:**
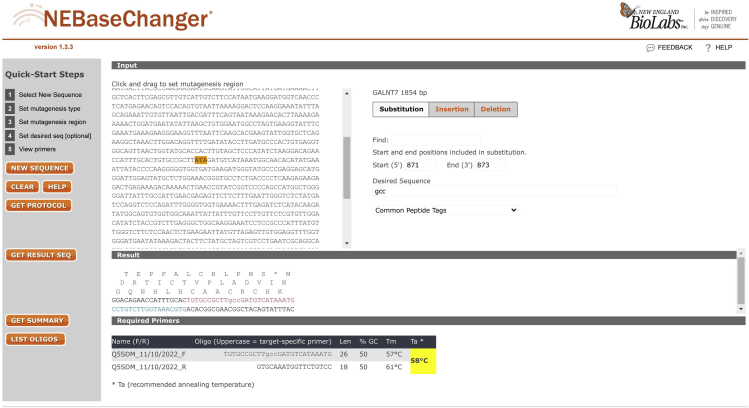
Representative image showing how to use NEBaseChanger to design the primers for site-directed mutagenesis of I330A from GalNAc-T7 The sequence of GalNAc-T7 is taken from the Insert Sequence of the GalNAc-T7 pGEN2-DEST plasmid from DNASU (http://dnasu.org/DNASU/GetCloneDetail.do?cloneid=413129).
4. Order the primers.
5. Prepare 10 μM solutions of the forward and reverse primers in nuclease-free water. Store at −20°C or −80°C.
  a. The primer solutions should be stable for months when stored at −20°C or −80°C.

***Note:*** To identify the mutagenesis region, the WT plasmid sequence can be copied into SnapGene. Since the amino acid sequence around the residues of interest is known (Figure 2), we can use the search functionality in SnapGene to find these residues and hence the start and end positions in the DNA sequence of the codon to be mutated.

3. Use NEBaseChanger™ (http://nebasechanger.neb.com/) to design the primers needed (Figure 4).
  a. Enter the WT plasmid DNA sequence.
  b. Select the mutagenesis type (Substitution).
  c. Select the mutagenesis region.
  d. Enter the new sequence.
  e. NEBaseChanger will provide the primer sequences together with the recommended annealing temperature to be used.

### Protein expression and Ni-NTA purification of WT and BH GalNAc-T

**Timing: 15 min**

6. Prepare fresh Wash Buffer: 20 mM imidazole, 50 mM Tris-HCl pH 7.5, 125 mM NaCl.
7. Prepare fresh Elution Buffer: 500 mM imidazole, 50 mM Tris-HCl pH 7.5, 125 mM NaCl.
8. Prepare fresh Freezing Buffer: 50 mM Tris-HCl pH 7.5, 125 mM NaCl, 20% (v/v) glycerol.

***Note:*** The buffers should be prepared fresh for the experiment. Any solution that isn’t used in the experiment can be discarded.

### *In vitro* glycosylation experiments

**Timing: 15 min**

9. Prepare 10× Buffer: 200 mM Tris-HCl pH 7.4, 500 mM NaCl and MilliQ water. Store at 20°C–25°C.
  a. The 10× Buffer should be stable for months when stored at 20°C–25°C.
10. Prepare 1× Buffer: 20 mM Tris-HCl pH 7.4, 50 mM NaCl and MilliQ water. Store at 20°C–25°C.
  a. The 1× Buffer should be stable for months when stored at 20°C–25°C.
11. Prepare a 100 mM MnCl_2_ solution in MilliQ water. Store at 20°C–25°C.
12. Prepare a 1 mM peptide solution in MilliQ water. Store at −20°C.
  a. Sonicate, taking care not to warm.
13. Prepare 5 mM solutions of UDP-GalNAc and compound **1** in MilliQ water. Store at −20°C or −80°C.
14. Prepare a 1 μM stock solution of WT and BH GalNAc-T in 1× Buffer.

**CRITICAL:** MnCl_2_ can be hazardous to human health. Always read the corresponding Material Safety Data Sheet (MSDS) before using this reagent and always handle it with care using appropriate personal protective cover (e.g., lab coat, gloves and safety goggles). Prepare the 1 μM stock solution of enzyme in 1× Buffer immediately before use and only prepare as much as you will need for the experiment. The enzyme cannot be stored in the 1× Buffer so any solution that is not used in the experiment should be discarded. The enzyme should always be handled on ice. Freeze-thawing cycles may affect the integrity of the enzyme. It is advisable to prepare aliquots with the volume required for the experiment to keep the freeze-thawing cycles to a minimum.

***Note:*** WT and BH GalNAc-T should be stable for months when stored at −80°C in Freezing Buffer. The peptide and UDP-sugar solutions can be stored for several months at −20°C but freeze-thawing may also affect their stability. It is therefore advisable to prepare aliquots with the volume required for the experiment to keep freeze-thawing cycles of the stock solutions to a minimum. Since MnCl_2_ can become oxidized a fresh solution should be made after 2 weeks to 1 month or before that if the solution turns brown.

### Michaelis-Menten kinetics

**Timing: 20 min**

15. Prepare 10× Buffer: 200 mM Tris-HCl pH 7.4, 500 mM NaCl and MilliQ water. Store at 20°C–25°C.
  a. The 10× Buffer should be stable for months when stored at 20°C–25°C.
16. Prepare 1× Buffer: 20 mM Tris-HCl pH 7.4, 50 mM NaCl and MilliQ water. Store at 20°C–25°C.
  a. The 1× Buffer should be stable for months when stored at 20°C–25°C.
17. Prepare a 100 mM MnCl_2_ solution in MilliQ water. Store at 20°C–25°C.
18. Prepare a 1 mM peptide solution in MilliQ water. Store at −20°C.
  a. Sonicate taking care not to warm.
19. Prepare 5 mM solutions of UDP-GalNAc and compound **1** in MilliQ water. Store at −20°C or −80°C.
  a. Prepare 10× solutions of UDP-GalNAc and compound **1** in MilliQ water by performing sequential dilutions from the 5 mM stock: 2,500 μM, 1,250 μM, 625 μM, 312.5 μM and 156.3 μM.
20. Prepare 10× stock solutions of WT and BH GalNAc-T in 1× Buffer: 20 nM, 40 nM, 80 nM, 160 nM, 320 nM, 640 nM, 1,250 nM and 2,500 nM.

**CRITICAL:** MnCl_2_ can be hazardous to human health. Always read the corresponding MSDS before using this reagent and always handle it with care using appropriate personal protective cover (e.g., lab coat, gloves and safety goggles). Prepare the 10× stock solutions of enzyme in 1× Buffer immediately before use and only prepare as much as you will need for the experiment. The enzyme cannot be stored in the 1× Buffer so any solution that isn’t used in the experiment should be discarded. The enzyme should always be handled on ice. Freeze-thawing cycles may affect the integrity of the enzyme. It is advisable to prepare aliquots with the volume required for the experiment to keep the freeze-thawing cycles to a minimum.

***Note:*** WT and BH GalNAc-T should be stable for months when stored at −80°C in Freezing Buffer. The peptide and UDP-sugar solutions can be stored for several months at −20°C but freeze-thawing may also affect their stability. It is therefore advisable to prepare aliquots with the volume required for the experiment to keep freeze-thawing cycles of the stock solutions to a minimum. Since MnCl_2_ can become oxidized a fresh solution should be made after 2 weeks to 1 month or before that if the solution turns brown.

### In-Fusion Cloning of full-length WT and BH GalNAc-T

**Timing: 5 min**

21. Design the PCR primers with 15 base pair (bp) extensions which are complementary to the ends of the pSBbi vector.
  a. We use the In-Fusion Cloning action in SnapGene to simplify PCR primer design (https://www.snapgene.com/resources/in-fusion-cloning/?referrer=SnapGene).
  b. In the Vector tab, open the file containing the desired pSBbi-based plasmid and select the option to linearize the vector with the SfiI restriction enzyme.
  c. In the Fragment tab, open the file containing the desired WT or BH GalNAc-T sequence.
    i. Highlight the region to be inserted.
    ii. Select the option to use the fragment as a template for PCR.
    iii. It is crucial to switch the orientation of the fragment by selecting the arrow pointing towards the left due to the nature of the pSBbi-based plasmids.
  d. In the Product tab, select the option to Choose Overlapping PCR Primers to allow SnapGene to design the most suitable primers for the experiment.
    i. Tick the option to regenerate the upstream and downstream SfiI sites from the Vector.
    ii. SnapGene will provide the primer sequences to be used in the In-Fusion cloning reaction and the sequence of the final product.
  e. Modify the sequence of the reverse primer provided by SnapGene so that a VSV-G tag is cloned at the C-terminus of the GalNAc-T followed by a stop codon.
    i. For example, the forward and reverse primers used for GalNAc-T2 were AAAGGCCTCTGAGGCCACCATGCGGCGGCGCGCTCG and TTTGGCCTGACAGGCCCTACTTACCCAGGCGGTTCATTTCGATATCAGTGTACTGCTGCAGGTTGAGCGGTG respectively (VSV-G tag underlined).^1^
22. Order the primers.
23. Prepare 10 μM solutions of the forward and reverse primers in nuclease-free water. Store at −20°C or −80°C.
  a. The primer solutions should be stable for months when stored at −20°C or −80°C.

### Cell transfection

**Timing: 15 min**

24. Prepare growth medium I: RPMI with 10% (v/v) FBS, 100 μg/mL penicillin and 100 μg/mL streptomycin. Store at 4°C.
25. Prepare growth medium containing 150 μg/mL hygromycin B. Store at 4°C.
26. Prepare growth medium containing 100 μg/mL hygromycin B. Store at 4°C.

**CRITICAL:** Reagents such as hygromycin B, penicillin and streptomycin can be hazardous to human health. Always read the corresponding MSDS before using these reagents and always handle them with care using appropriate personal protective cover (e.g., lab coat, gloves and safety goggles).

***Note:*** The growth medium with and without hygromycin B should be prepared fresh for the experiment and stored at 4°C for maximum a month, although it should be visually inspected every time it is used for contamination. If any contamination is observed the medium should be discarded and a fresh solution prepared.

### Cell surface labeling experiments

**Timing: 20 min**

27. Prepare 50 mM solutions of compound **2** and Ac_4_ManNAl in DMSO. Store at −80°C.
  a. The 50 mM solutions of compound **2** and Ac_4_ManNAl should be stable for months when stored at −80°C.
28. Prepare a 2% FBS in 1× PBS solution. Store at 4°C.
  a. The 2% FBS in 1× PBS should be stored at 4°C for maximum a month, although it should be visually inspected every time it is used for contamination.
  b. If any contamination is observed the medium should be discarded and a fresh solution prepared.
29. Prepare a 50 mM BTTAA solution in MilliQ water. Store at −20°C.
  a. The 50 mM BTTAA solution should be stable for months when stored at −20°C.
30. Prepare a 10 mM CF680 picolyl azide solution in DMSO. Store at −20°C.
  a. The 10 mM CF680 picolyl azide solution should be stable for months when stored at −20°C.
31. Prepare a fresh 30 mM CuSO_4_ solution in MilliQ water.
32. Prepare a fresh 100 mM sodium ascorbate solution in MilliQ water.
33. Prepare a fresh 100 mM aminoguanidinium chloride solution in 1× PBS.
34. Prepare a fresh 2× copper-catalyzed azide–alkyne cycloaddition (CuAAC) solution I as described in the “materials and equipment” section.
35. Prepare quenching solution: 3 mM bathocuproinedisulfonic acid in 1× PBS. Store at −20°C.
  a. The quenching solution should be stable for months when stored at −20°C.
36. Prepare fresh lysis buffer I as described in the “materials and equipment” section.
37. Prepare fresh diluted PNGase F solution: 1:10 (v/v) dilution in PBS.
38. Prepare 4× loading buffer: a 1:1:0.5:0.5 (v/v/v/v) mixture of 1 M Tris-HCl pH 7.0, 10% (w/v) SDS, 80% (v/v) glycerol and 1 M DTT. Store at −20°C.
  a. The 4× loading buffer should be stable for months when stored at −20°C.
39. Prepare fixing solution: 40% methanol and 10% acetic acid in MilliQ water. Store at 20°C–25°C.
  a. The fixing solution should be stable for months when stored at 20°C–25°C.

**CRITICAL:** Reagents such as aminoguanidinium chloride, CuSO_4_, DTT, and SDS can be hazardous to human health. Always read the corresponding MSDS before using these reagents and always handle them with care using appropriate personal protective cover (e.g., lab coat, gloves and safety goggles). Acetic acid and methanol are flammable and hazardous to human health. Keep these solvents away from ignition sources and always handle them in a chemical fume hood using appropriate personal protective cover. Prepare the diluted PNGase F solution immediately before use and only prepare as much as you will need for the experiment. Any solution that isn’t used in the experiment should be discarded. The enzyme should always be handled on ice.

***Note:*** The 30 mM CuSO_4_, the 100 mM sodium ascorbate and the 100 mM aminoguanidinium chloride solutions should be made fresh for each experiment. Any solution that isn’t used in the experiment should be discarded. PNGase F stock should be stable for months when stored at 4°C.

### Sample preparation for mass spectrometry (MS)-proteomics and glycoproteomics

**Timing: 3 h**

40. Prepare growth medium II: DMEM with 10% (v/v) FBS, 100 μg/mL penicillin and 100 μg/mL streptomycin. Store at 4°C.
  a. The growth medium should be prepared fresh for the experiment and stored at 4°C for maximum a month, although it should be visually inspected every time it is used for contamination.
  b. If any contamination is observed the medium should be discarded and a fresh solution prepared.
41. Prepare a 50 mM solution of compound **3** in DMSO. Store at −80°C.
  a. The 50 mM solution of compound **3** should be stable for months when stored at −80°C.
42. Prepare 8 mM EDTA in 1× PBS. Store at 20°C–25°C.
  a. The 8 mM EDTA in 1× PBS solution should be stable for months when stored at 20°C–25°C.
43. Prepare fresh lysis buffer II as described in the “materials and equipment” section.
44. Prepare a 50 mM BTTAA solution in MilliQ water. Store at −20°C.
  a. The 50 mM BTTAA solution should be stable for months when stored at −20°C.
45. Prepare a 10 mM Biotin-DADPS-picolyl azide solution in DMSO. Store at −20°C.
  a. The 10 mM Biotin-DADPS-picolyl azide solution should be stable for months when stored at −20°C.
46. Prepare a fresh 30 mM CuSO_4_ solution in MilliQ water.
47. Prepare a fresh 200 mM sodium ascorbate solution in MilliQ water.
48. Prepare a fresh 200 mM aminoguanidinium chloride solution in 1× PBS.
49. Prepare fresh 10× CuAAC solution II as described in the “materials and equipment” section.
50. Prepare cold methanol by storing it at −20°C until use.
51. Prepare 1% (w/v) RapiGest in 1× PBS. Store at −20°C.
  a. The 1% (w/v) Rapigest in 1× PBS solution should be stable for months when stored at −20°C.
52. Prepare 0.1% (w/v) RapiGest in 1× PBS. Store at −20°C.
  a. The 0.1% (w/v) Rapigest in 1× PBS solution should be stable for months when stored at −20°C.
53. Prepare fresh 6 M urea in 1× PBS. Store at 20°C–25°C.
  a. The 6 M urea in 1× PBS solution should be made fresh for the experiment and stored at 20°C–25°C for maximum a week as it can slowly decompose when in solution.
54. Prepare PBS-T: 0.1% Tween in 1× PBS. Store at 20°C–25°C.
  a. The PBS-T solution should be stable for months when stored at 20°C–25°C.
55. Prepare fresh Reagent A: 0.2 M sodium cyanoborohydride in PBS-T.
56. Prepare fresh Reagent B: 4% formaldehyde in PBS-T.
57. Prepare Lys-dimethylated Sera-Mag SpeedBeads Neutravidin-Coated Magnetic Beads^31^:
  a. Transfer beads to 15 mL Greiner tubes.
  b. Place the beads in a magnetic rack.
  c. Collect supernatant and store at 4°C until later (this buffer will be used once the methylation is complete to store the beads).
  d. Wash the beads three times with 10 mL of PBS-T.
  e. Place the beads in a magnetic rack.
  f. Add 5 mL of Reagent A and 5 mL of Reagent B to each sample and vortex.
  g. Leave the samples in the hood for 2 h with vortexing every 30 min.
  h. Place the beads in a magnetic rack.
  i. Discard supernatant.
  j. Add 1 M Tris-Cl pH 7 to the samples and vortex to quench any remaining reagents.
  k. Wash the beads three times with 10 mL of PBS-T.
  l. Resuspend the beads in the buffer the beads came in which was collected in step 57c.
  m. Store the beads at 4°C.
    i. The Lys-dimethylated Sera-Mag SpeedBeads Neutravidin-Coated Magnetic Beads are stable for months when stored at 4°C.^31^
58. Prepare fresh AmBic: 50 mM ammonium bicarbonate in LC/MS-grade water. Store at 4°C.
  a. The AmBic solution should be made fresh and stored for maximum a week at 4°C as the pH (which is optimum for trypsin activity) may deviate if the solution sits at 20°C–25°C.
59. Prepare fresh 40% (v/v) LC/MS-grade acetonitrile in LC/MS-grade water. Store at 4°C.
60. Prepare fresh 10 mM DTT in 50 mM AmBic.
  a. DTT is oxygen sensitive, therefore the solution should be made immediately before use.
61. Prepare fresh 20 mM iodoacetamide in 50 mM AmBic.
  a. Iodoacetamide is sensitive to light and not very stable in solution, therefore the solution should be made immediately before use and kept away from the light.
62. Prepare a 0.1 μg/μL Lys-C solution in LC/MS-grade water. Store at −80°C.
63. Prepare a 0.1 μg/μL trypsin solution in LC/MS-grade water. Store at −80°C.
64. Prepare fresh 1% (v/v) formic acid in LC/MS-grade water. Store at 4°C.
65. Prepare fresh conditioning solvent: 100% LC/MS-grade acetonitrile. Store at 4°C.
66. Prepare fresh loading buffer: 2% (v/v) LC/MS-grade acetonitrile/0.1% (v/v) formic acid in AmBic. Store at 4°C.
67. Prepare fresh wash buffer: 2% (v/v) LC/MS-grade acetonitrile in LC/MS-grade water. Store at 4°C.
68. Prepare fresh elution buffer: 80% (v/v) LC/MS-grade acetonitrile/0.1% (v/v) formic acid in LC/MS-grade water. Store at 4°C.

**Figure 5:**
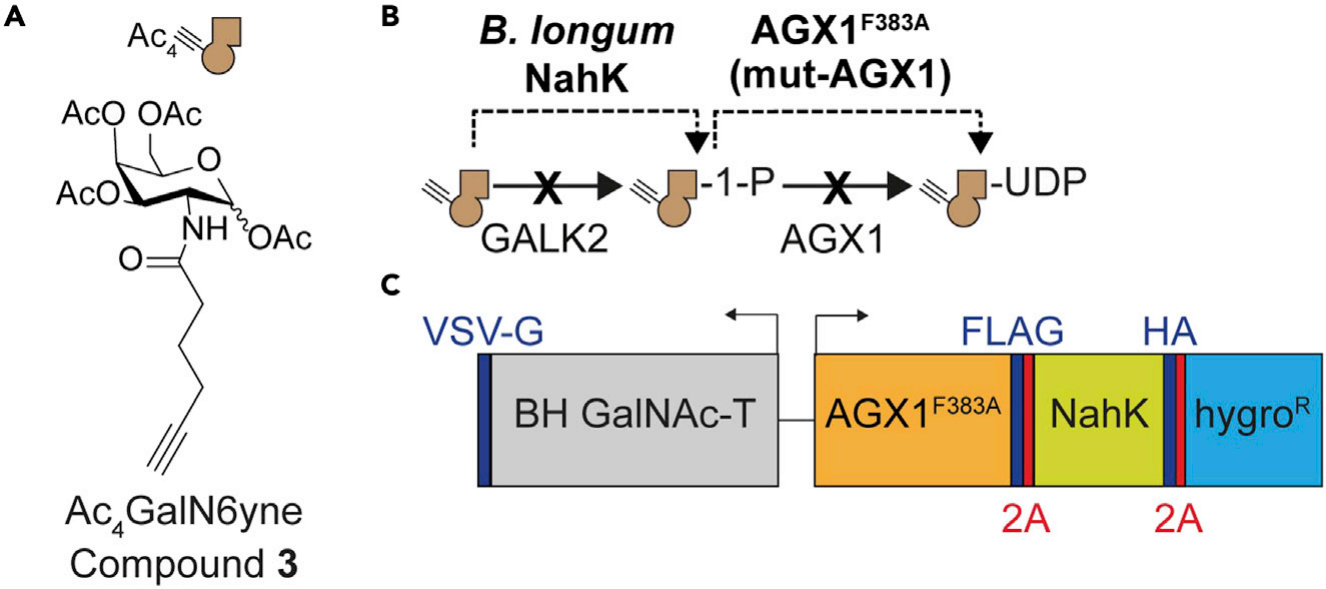
New BH engineering strategy for MS-glycoproteomics (A) In this new strategy, cells are treated with a membrane-permeable peracetylated GalNAc analogue precursor (Ac_4_GalN6yne, compound **3**) which is converted to the corresponding GalNAc analogue by esterases in the cell. (B) A promiscuous bacterial N-acetylhexosaminyl kinase (NahK) transforms the GalNAc analogue to the GalNAc-1-phosphate analogue which allows biosynthesis of the UDP-GalNAc analogue (compound **1**) by mut-AGX1. (C) BH GalNAc-T, mut-AGX1 and NahK co-expression construct required for this strategy to work in cells.

**CRITICAL:** Reagents such as aminoguanidinium chloride, AmBic, CuSO_4_ and DTT can be hazardous to human health. Always read the corresponding MSDS before using these reagents and always handle them with care using appropriate personal protective cover (e.g., lab coat, gloves and safety goggles). Acetonitrile, formaldehyde, formic acid, methanol and sodium cyanoborohydride are flammable and hazardous to human health. Keep these solvents away from ignition sources and always handle them in a chemical fume hood using appropriate personal protective cover.

***Note:*** Since the original publication, a new compound (Ac_4_GalN6yne, compound **3**) and a new construct containing an additional biosynthetic enzyme (N-acetylhexosamine 1-kinase (NahK) from *Bifidobacterium longum*) have been introduced that allow biosynthesis of compound **1** (Figure 5) in a more straightforward manner, as compound **3** is synthetically much more accessible than compound **2**^3^. Furthermore, the GalNAc-T-specific glycoprotein labeling and glycoproteomics workflow has been optimized to adherent MCF7 cells.^3^ The 30 mM CuSO_4_, the 200 mM sodium ascorbate and the 200 mM aminoguanidinium chloride solutions should be made fresh for each experiment. Any solution that isn’t used in the experiment can be discarded. The Reagent A and B solutions should be made fresh for the experiment and any solution that isn’t used should be discarded in the appropriate waste. The 0.1 μg/μL Lys-C and trypsin solutions in LC/MS-grade water should be stable for months when stored at −80°C. Since freeze-thawing cycles may affect the integrity of the enzymes, it is advisable to prepare aliquots with the volume required for the experiment and discard any solution that is not used. The 40% acetonitrile in LC/MS-grade water, 1% (v/v) formic acid in LC/MS-grade water, the conditioning solvent, the loading buffer, the wash buffer and the elution buffer should all be made fresh and stored at 4°C for maximum a week since acetonitrile and formic acid are volatile. These solutions should all be prepared in new clean glass vials since detergents and plastic leaching can cause background signal in the mass spectrometer and interfere with the analysis of the samples.

### Mass spectrometry data analysis

**Timing: 10 min**

69. Prepare a FASTA file containing the relevant protein sequences for your sample proteomes.
  a. If using human cells, download the *Homo sapiens* database from Uniprot or Swissprot.

## Key resources table

**Table:** 

REAGENT or RESOURCE	SOURCE	IDENTIFIER
**Antibodies**		

IRDye® 800CW Donkey anti-rabbit IgG (used at 1:10000)	LI-COR Biosciences (Lincoln, USA)	LI-COR Biosciences Cat# 926-32213; RRID AB_621848
Rabbit anti-FLAG (used at 1:1000)	Thermo Fisher Scientific (Waltham, USA)	Thermo Fischer Scientific Cat# PA1-984B; RRID: AB_347227
Rabbit anti-VSV-G (used at 1:500)	Thermo Fisher Scientific	Thermo Fischer Scientific Cat# PA1-29903; RRID: AB_1961363

**Chemicals, peptides, and recombinant proteins**		

Ac_4_GalN6yne (compound **3**)	Cioce et al.^3^	N/A
Ac_4_ManNAl	Click Chemistry Tools (Scottsdale, USA)	Click Chemistry Tools Cat# 1154-100; CAS No. 935658-93-8
Benzonase® Nuclease	Merck (Darmstadt, Germany)	Merck Cat# E1014-5KU; CAS No. 9025-65-4
Biotin-DADPS-picolyl azide	Schumann et al.^1^; subsequently custom synthesized from Sussex Research Laboratories (Ottawa, CA)	N/A
BTTAA	Jena Bioscience (Jena, Germany)	Jena Bioscience Cat# CLK-067-25; CAS No. 1334179-85-9
Bis(S-acetyl-2-thioethyl) 3,4,6-tri-O-acetyl-2-deoxy-2-(5-hexynoyl)amido-a-Dgalactopyranosyl phosphate (compound **2**)	Schumann et al.^1^	N/A
CF680 picolyl azide	Biotium (Fremont, USA)	Biotium Cat# 96003
CloneAmp™ HiFi PCR Premix	Takara Bio (Kusatsu, Japan)	Takara Cat# 639298
EA2 peptide PTTDSTTPAPTTK	AnaSpec (Fremont, USA)	AnaSpec Cat# AS-63841
Halt™ Protease Inhibitor Cocktail (100×)	Thermo Fisher Scientific	Thermo Fisher Scientific Cat# 1861279
Intercept (TBS) Blocking Buffer	LI-COR Biosciences	LI-COR Biosciences Cat# 927-60001
IRDye® 800CW Streptavidin (used at 1:5000)	LI-COR Biosciences	LI-COR Biosciences Cat# 926-32230
Lipofectamine™ LTX Reagent with PLUS™ Reagent	Thermo Fisher Scientific	Thermo Fisher Scientific Cat# 15338100
Lys-C, Mass Spec Grade	Promega (Madison, USA)	Promega Cat# VA1170
Ni-NTA® Agarose beads	Qiagen (Hilden, Germany)	Qiagen Cat# 30210
Nuclease free water	Thermo Fisher Scientific	Thermo Fischer Scientific Cat# AM9937
Opti-MEM® I Reduced Serum Medium	Thermo Fisher Scientific	Thermo Fischer Scientific Cat# 31985062
PNGase F	Promega	Promega Cat# V4831A
PUGNAc	Sigma-Aldrich (St. Louis, USA)	Sigma Cat# A7229; CAS No. 132489-69-1
RapiGest SF	Waters (Milford, USA)	Waters Cat# 186002122
SafeBlue Protein Stain	NBS Biologicals (Huntingdon, UK)	NBS Biologicals Cat# NBS-SB1L
Sera-Mag SpeedBeads Neutravidin-Coated Magnetic Beads	Cytiva (Marlborough, USA)	Cytiva Cat# 78152104011150
SfiI restriction enzyme	New England Biolabs (Ipswich, USA)	New England Biolabs Cat# R0123S
Soluble His_6_-tagged BH-T2 protein	Schumann et al.^1^	N/A
Trypsin Gold, mass spectrometry grade	Promega	Promega Cat# V528A
Uridine 5′-diphospho-N-acetylgalactosamine disodium salt (UDP-GalNAc)	Sigma-Aldrich	Sigma Aldrich Cat# U5252; CAS No. 108320-87-2
Uridine 5′-diphospho-2-deoxy-2-(5-hexynoyl)amido-α-D-galactopyranoside disodium salt (compound **1**)	Choi et al.^12^	N/A

**Critical commercial assays**		

ExpiFectamine™ 293 Transfection Kit	Thermo Fischer Scientific	Thermo Fischer Scientific Cat# A14525
In-Fusion HD Cloning Kit	Takara Bio	Takara Bio Cat# 102518
NucleoSpin® Gel and PCR Clean-up Kit	Macherey-Nagel (Düren, Germany)	Macherey-Nagel Cat# 740609.50
Pierce™ BCA Protein Assay Kit	Thermo Fischer Scientific	Thermo Fischer Scientific Cat# 23227
Q5® Site-Directed Mutagenesis Kit	New England Biolabs	New England Biolabs Cat# E0554S
QIAprep Spin Miniprep Kit	Qiagen	Qiagen Cat# 27106
Revert™ 700 Total Protein Stain Kit	LI-COR Bioscience	LI-COR Bioscience Cat# 926-11016
Trans-Blot Turbo RTA Midi 0.2 μm Nitrocellulose Transfer Kit	Bio-Rad Laboratories (Hercules, USA)	Bio-Rad Laboratories Cat# 1704271
ZymoPURE™ II Plasmid Maxiprep Kit	Zymo Research (Irvine, USA)	Zymo Research Cat# D4203

**Deposited data**		

Crystal structure BH-T2/EA2/UDP/Mn2+	Schumann et al.^1^	PDB: 6E7I
Crystal structure BH-T2/compound **1**/Mn2+	Schumann et al.^1^	PDB: 6NQT
Gel and blot full images	Schumann et al.^1^	https://doi.org/10.17632/nh4vww6hxj.2
Glycoproteomics raw data	Schumann et al.^1^	PRIDE accession ID: PXD018048
Proteomics raw data	Schumann et al.^1^	PRIDE accession ID: PXD017989

**Experimental models: Cell lines**		

Expi293F™ cells	Thermo Fischer Scientific	Thermo Fischer Scientific Cat# A14527
K-562	Laboratory of Jonathan Weissman, UCSF	N/A
K-562 pSBbi-WT-hAGX1-BH-T1	Schumann et al.^1^	N/A
K-562 pSBbi-mut-hAGX1-WT-T1	Schumann et al.^1^	N/A
K-562 pSBbi-mut-hAGX1-BH-T1	Schumann et al.^1^	N/A
K-562 pSBbi-WT-hAGX1-BH-T2	Schumann et al.^1^	N/A
K-562 pSBbi-mut-hAGX1-WT-T2	Schumann et al.^1^	N/A
K-562 pSBbi-mut-hAGX1-BH-T2	Schumann et al.^1^	N/A
MCF7 cells	ATCC (Manassas, USA)	ATCC Cat# HTB-22; RRID: CVCL_0031
MCF7 pSBbi-mut-hAGX1-NahK-BH T2	Cioce et al.^3^	N/A
Stellar™ Competent Cells	Takara Bio	Takara Bio Cat# 636763

**Oligonucleotides**		

T1 and T2 site-directed mutagenesis primers	Choi et al.^12^	N/A
T1 and T2 In-Fusion primers	Schumann et al.^1^	N/A

**Recombinant DNA**		

pCMV(CAT)T7-SB100	Addgene (Watertown, USA)	Addgene Cat# 34879; RRID: Addgene_34879
pDONR221	Thermo Fischer Scientific	Thermo Fischer Scientific Cat# 12536017
pGEN2-DEST	Moremen et al.^30^	N/A
pOPING-trunc-BH-T2	Schumann et al.^1^	N/A
pSBbi-WT-hAGX1-BH-T1	Schumann et al.^1^	N/A
pSBbi-mut-hAGX1-WT-T1	Schumann et al.^1^	N/A
pSBbi-mut-hAGX1-BH-T1	Schumann et al.^1^	N/A
pSBbi-WT-hAGX1-BH-T2	Schumann et al.^1^	N/A
pSBbi-mut-hAGX1-WT-T2	Schumann et al.^1^	N/A
pSBbi-mut-hAGX1-BH-T2	Schumann et al.^1^	N/A

**Software and algorithms**		

Byonic	Protein Metrics (Cupertino, USA)	N/A
GraphPad Prism (non-linear Michaelis-Menten and kcat fitting programs)	Dotmatics (Boston, USA)	N/A
Image Studio	LI-COR Bioscience	N/A
Perseus	Tyanova et al.^32^	N/A
PyMOL	Schrödinger, Inc. (New York, USA)	N/A
MaxQuant	Cox et al.^33^	N/A
SnapGene	Dotmatics	N/A

**Other**		

4%–20% Criterion™ TGX™ Precast Midi Protein Gel, 12+2 well, 45 μL	Bio-Rad Laboratories	Bio-Rad Laboratories Cat# 5671093
4%–20% Criterion™ TGX™ Precast Midi Protein Gel, 18 well, 30 μL	Bio-Rad Laboratories	Bio-Rad Laboratories Cat# 5671094
4%–20% Criterion™ TGX™ Precast Midi Protein Gel, 26 well, 15 μL	Bio-Rad Laboratories	Bio-Rad Laboratories Cat# 5671095
Steritop Threaded Bottle Top Filter	Sigma-Aldrich	Sigma Aldrich Cat# S2GPT01RE
Amicon Ultra-15 Centrifugal Filters (3 kDa MWCO)	Merck	Merck Cat# UFC900308
Protein LoBind® Tubes, 1.5 mL	Eppendorf (Hamburg, Germany)	Eppendorf Cat# 0030108116
Nalgene™ 0.2 μM PES syringe filter	Thermo Fisher Scientific	Thermo Fisher Scientific Cat# 720-1320
Odyssey CLx	LI-COR Bioscience	LI-COR Bioscience Model # 9140
SnakeSkin™ Dialysis Tubing, 10K MWCO, 16 mm	Thermo Fisher Scientific	Thermo Fisher Scientific Cat# 88243
UltraMicroSpin™ columns	The Nest group, Inc. (Ipswich, USA)	The Nest group, Inc. Cat# SUM SS18V

## Materials and equipment

**Table:** 2× CuAAC solution I

Reagent	Stock concentration	Desired concentration in 2× CuAAC solution	Final concentration in samples (1×)	Amount (μL)
BTTAA	50 mM	1.2 mM	600 μM	12
CuSO_4_	30 mM	200 μM	100 μM	3.3
Sodium ascorbate	100 mM	5 mM	2.5 mM	25
Aminoguanidinium chloride	100 mM	5 mM	2.5 mM	25
CF680 picolyl azide	10 mM	200 μM	100 μM	10
MilliQ water	N/A	N/A	N/A	424.7
**Total**	**N/A**	**N/A**	**N/A**	**500**

**CRITICAL:** Reagents such as aminoguanidinium chloride and CuSO_4_ can be hazardous to human health. Always read the corresponding MSDS before using these reagents and always handle them with care using appropriate personal protective cover (e.g., lab coat, gloves and safety goggles). The 2× CuAAC solution I should be prepared by adding the components in the order of the table.

**Table:** Lysis buffer I

Reagent	Stock concentration	Final concentration	Amount (μL)
Tris-HCl pH 8.0	1 M	50 mM	75
NaCl	5 M	150 mM	45
Triton X-100	20%	1%	75
Sodium deoxycholate	10%	0.5%	75
SDS	20%	0.1%	7.5
MgCl_2_	1 M	1 mM	1.5
Benzonase nuclease	250 U/μL	100 mU/μL	0.6
Halt Protease Inhibitors	100×	1×	15
MilliQ water	N/A	N/A	1205.4
**Total**	**N/A**	**N/A**	**1500**

**CRITICAL:** Reagents such as Halt Protease Inhibitors, Triton X-100, sodium deoxycholate and SDS can be hazardous to human health. Always read the corresponding MSDS before using these reagents and always handle them with care using appropriate personal protective cover (e.g., lab coat, gloves and safety goggles). The benzonase nuclease and Halt protease inhibitors must be added to the lysis buffer immediately before adding the lysis buffer to the cells. Only prepare as much lysis buffer I as you will need for the experiment. Any solution that isn’t used in the experiment should be discarded.

***Note:*** Lysis buffer containing all reagents except benzonase nuclease and Halt Protease inhibitors can be prepared and stored at 4°C in the dark (since sodium deoxycholate is light sensitive) for a couple of months or until some precipitate is observed.

**Table:** Lysis buffer II

Reagent	Stock concentration	Final concentration	Amount (μL)
Tris-HCl pH 8.0	1 M	50 mM	75
NaCl	5 M	150 mM	45
Triton X-100	20%	1%	75
Sodium deoxycholate	10%	0.5%	75
SDS	20%	0.1%	7.5
MgCl_2_	1 M	1 mM	1.5
Benzonase nuclease	250 U/μL	100 mU/μL	0.6
Halt Protease Inhibitors	100×	1×	15
PUGNAc	50 mM	50 μM	1.5
MilliQ water	N/A	N/A	1203.9
**Total**	**N/A**	**N/A**	**1500**

**CRITICAL:** Reagents such as Halt Protease Inhibitors, Triton X-100, sodium deoxycholate and SDS can be hazardous to human health. Always read the corresponding MSDS before using these reagents and always handle them with care using appropriate personal protective cover (e.g., lab coat, gloves and safety goggles). The benzonase nuclease, Halt protease inhibitors and PUGNAc must be added to the lysis buffer immediately before adding the lysis buffer to the cells. Only prepare as much lysis buffer II as you will need for the experiment. Any solution that isn’t used in the experiment should be discarded.

***Note:*** Lysis buffer containing all reagents except benzonase nuclease, Halt Protease inhibitors and PUGNAc can be prepared and stored at 4°C in the dark (since sodium deoxycholate is light sensitive) for a couple of months or until some precipitate is observed. The difference between lysis buffer I and lysis buffer II is the addition of PUGNAc (which is an O-GlcNAcase and β-hexosaminidase inhibitor) to the latter.

**Table:** 10× CuAAC solution II

Reagent	Stock concentration	Desired concentration in 10× CuAAC solution	Final concentration in samples (1×)	Amount (μL)
BTTAA	50 mM	6 mM	600 μM	60
CuSO_4_	30 mM	3 mM	300 μM	50
Sodium ascorbate	200 mM	50 mM	5 mM	125
Aminoguanidinium chloride	200 mM	50 mM	5 mM	125
Biotin-DADPS-picolyl azide	10 mM	1 mM	100 μM	50
MilliQ water	N/A	N/A	N/A	90
**Total**	**N/A**	**N/A**	**N/A**	**500**

**CRITICAL:** Reagents such as aminoguanidinium chloride and CuSO_4_ can be hazardous to human health. Always read the corresponding MSDS before using these reagents and always handle them with care using appropriate personal protective cover (e.g., lab coat, gloves and safety goggles). The 10× CuAAC solution II should be prepared by adding the components in the order of the table.

***Note:*** The differences between CuAAC solution I and CuAAC solution II are the different concentrations of the reagents and the different azide-containing probes used (CF680 picolyl azide in the former and Biotin-DADPS-picolyl azide in the latter).

## Step-by-step method details

### Cloning of BH GalNAc-Ts

**Timing: 2 weeks**

This step is performed to replace the gatekeeper residues in the active site of the GalNAc-T with alanines and thus generate the BH GalNAc-T. This is performed on both the full-length and truncated constructs of the GalNAc-T, with the two-point mutations introduced in a sequential manner.

1. Site directed mutagenesis (troubleshooting 1 and 2).
  a. For this step we use the Q5® Site-Directed Mutagenesis Kit according to the manufacturer’s instructions (https://international.neb.com/protocols/2013/01/26/q5-site-directed-mutagenesis-kit-protocol-e0554).
    i. It is advisable to run the PCR product on an agarose gel to confirm the success of the PCR reaction prior to the KLD treatment.
2. Purify plasmid DNA by Miniprep.
  a. Pick a single colony from the transformed bacteria and inoculate a 3 mL culture of LB medium containing the appropriate selection antibiotic.
    i. The tip used to pick the bacterial colony can be used to streak an appropriate selection agar plate before placing the tip into the LB culture to have a backup.
  b. Incubate at 37°C for at least 6 h (preferably for 16–20 h) in a shaking incubator at >200 rpm.
  c. Transfer 2 mL of the culture into a 15 mL centrifuge tube and centrifuge at >10,000 × *g* for 5 min at 20°C–25°C.
  d. We use the QIAprep Spin Miniprep Kit and a microcentrifuge to purify and isolate the plasmid DNA according to the manufacturer’s instructions (https://www.qiagen.com/us/resources/download.aspx?id=22df6325-9579-4aa0-819c-788f73d81a09&lang=en).
3. Confirm the presence of the point mutation and the absence of any undesired mutations by Sanger Sequencing.
4. Repeat steps 1–3 to introduce the second point mutation.
5. Once the presence of both mutations has been confirmed by Sanger Sequencing, use a positive colony to inoculate a 100 mL culture of LB medium containing the appropriate selection antibiotic.
6. Incubate at 37°C for at least 6 h (preferably for 16–20 h) in a shaking incubator at >200 rpm.
7. Purify plasmid DNA by Maxiprep.
  a. We use the ZymoPURE™ II Plasmid Maxiprep Kit and a vacuum manifold according to the manufacturer’s instructions (https://files.zymoresearch.com/protocols/_d4202_d4203_zymopure_ii_plasmid_maxiprep.pdf).

**Pause point:** The PCR products can be stored at −20°C before performing the KLD reaction. The KLD reaction products can be stored at −20°C before performing the transformation step.

***Note:*** We recommend using the pDONR221 and pGEN2-DEST vectors for the full-length and truncated constructs respectively which are commercially available in DNASU. We have observed that the mutagenesis efficiency strongly depends on the vector used therefore if alternative constructs are used the mutagenesis protocol may need to be optimized accordingly.

### Protein expression and Ni-NTA purification of WT and BH GalNAc-T

**Timing: 2 weeks**

This step is performed to express purified recombinant His-tagged truncated WT and BH GalNAc-Ts.

8. Express His-tagged truncated WT and BH GalNAc-T in Expi293F™ cells (troubleshooting 3).
  a. Transfect the cells with the expression plasmids coding His-tagged truncated WT and BH GalNAc-T using the ExpiFectamine™ 293 Transfection Kit according to the manufacturer’s instructions (https://www.thermofisher.com/document-connect/document-connect.html?url=https://assets.thermofisher.com/TFS-Assets%2FLSG%2Fmanuals%2FMAN0007814_Expi293_ExpressionSystem_UG.pdf).
  b. After four days centrifuge the cells at 200 × *g* for 5 min, collect and filter the supernatant.
    i. We use Steritop Threaded Bottle Top Filters to filter the collected supernatant.
9. Purify His-tagged truncated WT and BH GalNAc-T by Ni-NTA purification.
  a. Add 1 mM NiSO_4_ to the collected supernatant.
    i. This step is performed to prevent non-specific binding to the beads.
  b. Incubate for 15 min at 4°C on a tube roller shaker.
  c. Condition Ni-NTA® Agarose beads three times with 5 column volumes (CV) of water and twice with 5 CV of Wash buffer.
  d. Add the supernatant to the beads.
  e. Incubate the samples for 16–20 h at 4°C on a tube roller shaker.
  f. Wash the beads three times with 10 CV of Wash Buffer.
  g. Elute the protein by adding 2 CV of:
    i. 20% Elution Buffer (100 mM imidazole = 4/5 Wash Buffer and 1/5 Elution Buffer).
    ii. 40% Elution Buffer (200 mM imidazole = 3/5 Wash Buffer and 2/5 Elution Buffer).
    iii. 40% Elution Buffer (200 mM imidazole = 3/5 Wash Buffer and 2/5 Elution Buffer).
    iv. 60% Elution Buffer (300 mM imidazole = 2/5 Wash Buffer and 3/5 Elution Buffer).
  h. Dialyze the eluted protein for 16–20 h using SnakeSkin™ Dialysis Tubing against Freezing Buffer.
  i. Determine the protein concentration.
  j. Aliquot, flash-freeze in liquid nitrogen and store at −80°C.
    i. WT and BH GalNAc-T should be stable for months when stored at −80°C in Freezing Buffer.

***Note:*** We recommend determining the protein concentration by densitometry of Coomassie-stained SDS-PAGE gel bands and comparison to known standards of bovine serum albumin since certain components in the enzyme preparation (e.g., imidazole) may interfere with measurements performed by BCA assay or Nanodrop.

### *In vitro* glycosylation experiments

**Timing: 2 days**

This step assesses and compares the enzymatic activities of the WT and BH systems. To ensure successful bump-and-hole engineering, the BH GalNAc-T must remain biochemically competent whilst orthogonal to the WT enzyme. Moreover, to ensure that bump-and-hole engineering does not affect the enzyme’s peptide substrate affinity and specificity this experiment should also be carried out with a small panel of synthetic peptides.

10. Mix the following reagents in PCR tubes:

**Table:** 

Reagent	Stock concentration	Final concentration	Volume (μL)
10× Buffer	10×	1×	2.5
MnCl_2_	100 mM	10 mM	2.5
UDP-sugar	5 mM	250 μM	1.25
Peptide	1 mM	50 μM	1.25
WT/BH GalNAc-T	1 μM	100 nM	2.5
MilliQ water	N/A	N/A	15
**Total**	**N/A**	**N/A**	**25**

**Figure 6:**
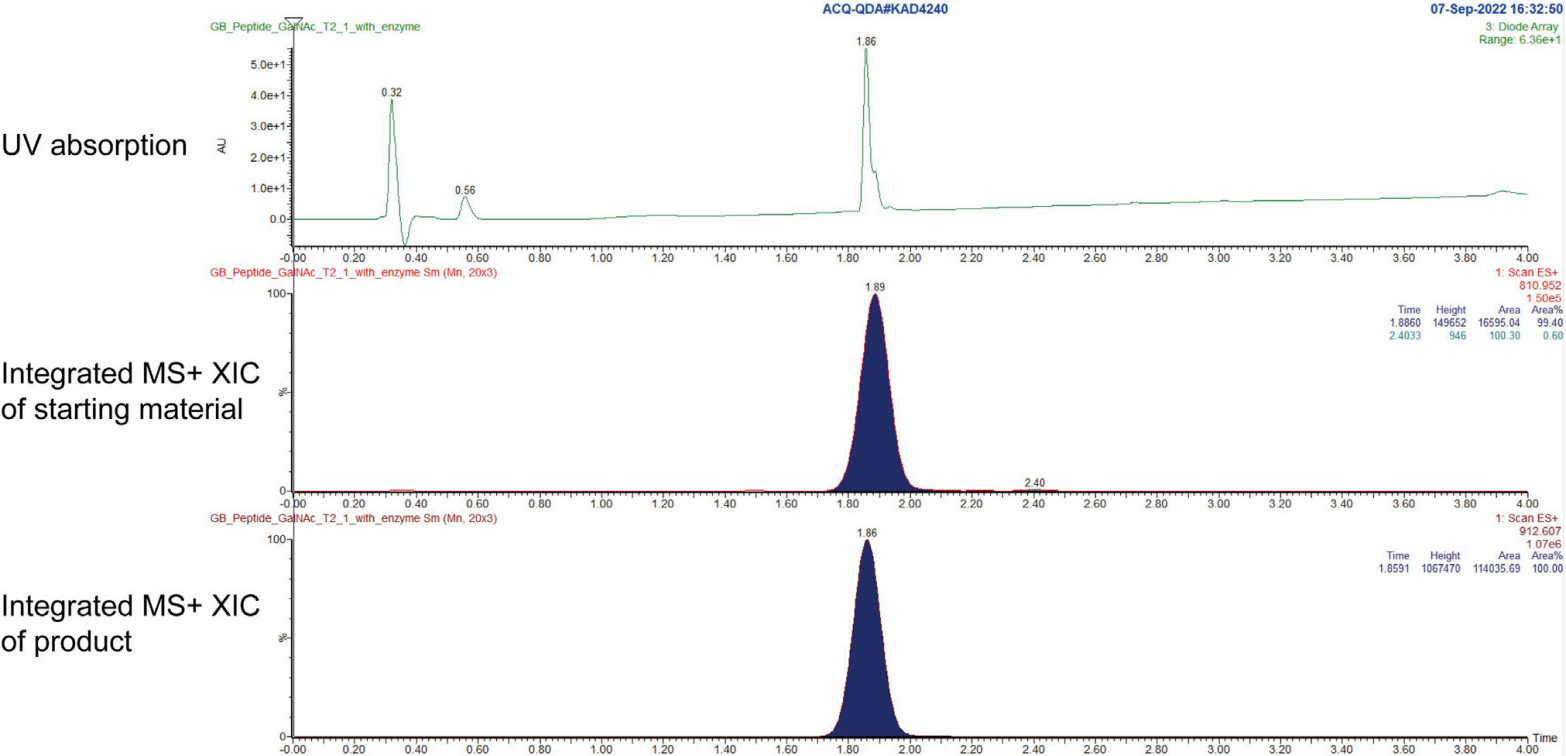
Representative UV and MS traces of the starting material (810.9 m/z, 1.5 × 10^5^ a.u.) and product (912.6 m/z, 1.07 × 10^6^ a.u.) following an *in vitro* glycosylation experiment between WT GalNAc-T2 and UDP-GalNAc Based on signal integration, the conversion in this experiment was calculated as 87.7%. XIC = extracted ion chromatogram.

11. Incubate at 37°C for 16–20 h.
12. Quench by heating at 95°C for 10 s before placing on ice.
13. Determine glycopeptide formation by HPLC-MS and MS+ peak integration (Figure 6) using Equation 1.

(Equation 1) $$Glycopeptide\;formation\left(\%\right)=\frac{Peak\;area\;of\;product}{Peak\;area\;of\;starting\;material+Peak\;area\;of\;product}\times 100$$

**CRITICAL:** Always handle the enzyme on ice and add it to the reaction mixture last.

***Optional:*** A fluorescently-labelled synthetic peptide may be used instead, followed by determination of glycopeptide formation by UV peak integration. However, the chromatography method may need to be optimized to separate the peptide starting material and glycopeptide product peaks. The UDP-Glo^TM^ Glycosyltransferase Assay may also be used as an alternative way of quantifying the activity of the enzymes, however it will not take into account UDP-sugar hydrolysis which may skew the results.

***Note:*** The activity of the enzymes may vary between GalNAc-Ts and enzyme preparations. The conditions of the glycosylation reaction may therefore need to be optimized accordingly. We have demonstrated that accurate quantification by MS+ peak integration is possible due to comparable ionization efficiencies of the peptide starting material and glycopeptide product. Nevertheless, since ion counts might differ between (glyco-)peptides it is recommended that whenever a new (glyco-)peptide is used that the ion count is compared against the native enzyme-substrate pair to confirm that this is the case. We have previously demonstrated that compound **1** is compatible with several GalNAc-Ts for the BH approach in that orthogonal enzyme activity is observed for compound **1** as a substrate (activity is observed with BH GalNAc-T only, with comparable glycopeptide turnover to the native system).^12^ However, alternative UDP-GalNAc analogues may be investigated for other GalNAc-T systems.

### Michaelis-Menten kinetics

**Timing: 2 days**

This step confirms whether the BH GalNAc-T/compound **1** pair retains the kinetic parameters of its WT GalNAc-T/UDP-GalNAc counterpart. First, the enzyme concentration at which ∼10%–20% glycopeptide formation is achieved needs to be determined and this value is chosen to fulfill the prerequisites of the Michaelis-Menten kinetics.

14. Set up *in vitro* glycosylation reactions using different WT and BH GalNAc-T concentrations: 2 nM, 4 nM, 8 nM, 16 nM, 32 nM, 64 nM.
  a. For less active enzymes, higher enzyme concentrations might be needed.

**Table:** 

Reagent	Stock concentration	Final concentration	Volume (μL)
10× Buffer	10×	1×	2.5
MnCl_2_	100 mM	10 mM	2.5
UDP-sugar	5 mM	250 μM	1.25
Peptide	1 mM	50 μM	1.25
WT/BH GalNAc-T	10×	1×	2.5
MilliQ water	N/A	N/A	15
**Total**	**N/A**	**N/A**	**25**

15. Incubate at 37°C for 1.5 h.
16. Quench by heating at 95°C for 10 s before placing on ice.
17. Determine glycopeptide formation by HPLC-MS and MS+ peak integration using Equation 1.
18. Determine the enzyme concentration at which ∼10%–20% glycopeptide formation is obtained (starting kinetics).
19. Set up *in vitro* glycosylation reactions using the determined enzyme concentration and different UDP-sugar concentrations: 15.6 μM, 32.2 μM, 62.5 μM, 125 μM, 250 μM, 500 μM.
  a. Increase the peptide concentration to at least twice the Km to ensure that it is present in excess.

**Table:** 

Reagent	Stock concentration	Final concentration	Volume (μL)
10× Buffer	10×	1×	2.5
MnCl_2_	100 mM	10 mM	2.5
UDP-sugar	10×	1×	2.5
Peptide	1 mM	250 μM	6.25
WT/BH GalNAc-T	10×	1×	2.5
MilliQ water	N/A	N/A	8.75
**Total**	**N/A**	**N/A**	**25**

20. Incubate at 37°C for 1.5 h.
21. Quench by heating at 95°C for 10 s before placing on ice.
22. Determine glycopeptide formation by HPLC-MS and MS+ peak integration using Equation 1.
23. Divide the measured glycopeptide formation over the duration of the reaction (5400 s) to calculate the initial rate of reaction at each substrate concentration.
24. Determine the kinetic parameters for the BH GalNAc-T/compound **1** and WT GalNAc-T/UDP-GalNAc pairs.
  a. We use the non-linear Michaelis-Menten and kcat fitting programs found in GraphPad Prism (https://www.graphpad.com/guides/prism/latest/curve-fitting/reg_michaelis_menten_enzyme.htm).

**CRITICAL:** Always handle the enzyme on ice and add it to the reaction mixture last. If any of the requirements of Michaelis-Menten kinetics are not fulfilled, the model will be unsuitable. Furthermore, not all enzymes adhere to Michaelis-Menten kinetics. The Michaelis-Menten equation describes a rectangular hyperbola. If the plot of initial rate of reaction against substrate concentration does not display this behavior, alternative kinetic models may be more suitable.

***Note:*** Higher WT and BH GalNAc-T enzyme concentrations can be investigated. The peptide concentration used in the experiment using different UDP-sugar concentrations will depend on the Km of the GalNAc-T with the peptide used. We chose 1.5 h as a convenient endpoint to measure glycopeptide formation at the initial stage of the reaction. Alternative timepoints may be used instead as long as ∼10% glycopeptide formation is obtained. Please refer to Choi et al.^12^ for examples of Michaelis-Menten kinetics plots of WT and BH enzyme-substrate pairs for GalNAc-T1, -T2 and -T10.

### In-Fusion Cloning of full-length WT and BH GalNAc-T into pSBbi plasmids

**Timing: 1 week**

This step is performed to clone full-length WT and BH GalNAc-T with a C-terminal VSV-G tag into the backbone of pSBbi-based plasmids containing FLAG-tagged WT/mut-AGX1.

25. Prepare a linearized vector by restriction digestion.
  a. The restriction enzyme we use is SfiI according to the manufacturer’s instructions (http://nebcloner.neb.com/#!/protocol/re/single/SfiI).
26. Amplify the target fragment (i.e., WT/BH GalNAc-T) by PCR.
  a. We use the CloneAmp HiFi PCR Premix according to the manufacturer’s instructions (https://www.takarabio.com/documents/User%20Manual/CloneAmp%20HiFi%20PCR%20Premix%20Protocol/CloneAmp%20HiFi%20PCR%20Premix%20Protocol-At-A-Glance_092612.pdf).
27. Analyze the linearized vector and PCR product by agarose gel electrophoresis to confirm that a single band is seen at the expected molecular weight.
28. Purify the linearized vector and the PCR products by gel extraction.
  a. For this step we use the NucleoSpin® Gel and PCR Clean-up Kit according to the manufacturer’s instructions (https://www.takarabio.com/documents/User%20Manual/NucleoSpin%20Gel%20and%20PCR%20Clean/NucleoSpin%20Gel%20and%20PCR%20Clean-up%20User%20Manual_Rev_04.pdf).
29. Set up the In-Fusion cloning reaction.
  a. We use the In-Fusion® HD Cloning kit according to the manufacturer’s instructions (https://takara.co.kr/file/manual/pdf/pt5162-1.pdf).
  b. Takara’s In-Fusion molar ratio calculator can be used to calculate the optimal amounts of vector and insert to use in the In-Fusion Cloning reaction (https://www.takarabio.com/learning-centers/cloning/primer-design-and-other-tools/in-fusion-molar-ratio-calculator).
30. Transform the cloning reaction products.
  a. We use Stellar™ Competent Cells according to the manufacturer’s instructions (https://www.takarabio.com/documents/User%20Manual/PT5055/PT5055-2.pdf).
31. Purify plasmid DNA by Miniprep.
  a. Pick a single colony from the transformed bacteria and inoculate a 3 mL culture of LB medium containing the appropriate selection antibiotic.
    i. The tip used to pick the bacterial colony can be used to streak an appropriate selection agar plate before placing the tip into the LB culture to have a backup.
  b. Incubate at 37°C for at least 6 h (preferably 16–20 h) in a shaking incubator at >200 rpm.
  c. Transfer 2 mL of the culture into a 15 mL centrifuge tube and centrifuge at >10000 × *g* for 5 min at 20°C–25°C.
  d. We use the QIAprep Spin Miniprep Kit and a microcentrifuge to purify and isolate the plasmid DNA according to the manufacturer’s instructions (https://www.qiagen.com/us/resources/download.aspx?id=22df6325-9579-4aa0-819c-788f73d81a09&lang=en).
32. Confirm the success of the In-Fusion Cloning reaction by Sanger Sequencing.
33. Once the success of the reaction has been confirmed, use a positive colony to inoculate a 100 mL culture of LB medium containing the appropriate selection antibiotic.
34. Incubate at 37°C for at least 6 h (preferably for 16–20 h) in a shaking incubator at >200 rpm.
35. Purify plasmid DNA by Maxiprep.
  a. We use the ZymoPURE™ II Plasmid Maxiprep Kit and a vacuum manifold according to the manufacturer’s instructions (https://files.zymoresearch.com/protocols/_d4202_d4203_zymopure_ii_plasmid_maxiprep.pdf).

**Pause point:** The purified linearized vector and PCR products can be stored at –20°C prior to the In-Fusion cloning reaction. The cloning reactions can be stored at –20°C prior to the transformation step.

### Cell transfection

**Timing: 2 weeks**

This step is performed to establish stable K-562 cell lines using the pSBbi plasmids co-expressing VSV-G-tagged WT/BH GalNAc-T and FLAG-tagged WT/mut-AGX1. These plasmids also contain a hygromycin resistance gene to allow the generation of stable colonies through hygromycin selection (Figure 1). We co-transfect the pSBbi plasmids with a Sleeping Beauty transposase for stable integration of the plasmid DNA into the genome of the K-562 cells.

36. Plate K-562 cells at 70%–90% confluency in 1.5 mL of growth medium in a 6-well plate.
37. Transfect the cells with Lipofectamine LTX according to the manufacturer’s instructions (https://tools.thermofisher.com/content/sfs/manuals/LipofectamineLTX_PLUS_Reag_protocol.pdf) using 2.4 μg pSBbi plasmid and 125 ng pCMV(CAT)T7-SB100 plasmid DNA per well.
  a. Include the following controls:
    i. Control of transient vs stable expression using 2.4 μg pSBbi plasmid but no pCMV(CAT)T7-SB100 plasmid DNA.
    ii. Control with no plasmid DNA – these cells should die as soon as they are treated with hygromycin B due to the lack of a hygromycin resistance gene.
38. Incubate cells at 37°C for 16–20 h.
39. Harvest the cells and resuspend in 2 mL of growth medium containing 150 μg/mL hygromycin B.
40. Maintain selection for 7–10 days to establish stable colonies.
  a. Change to fresh media containing 150 μg/mL hygromycin B every two days.
  b. Once the controls have died, the hygromycin B concentration in the growth medium can be reduced to 100 μg/mL.

***Note:*** We prepare the diluted Lipofectamine LTX solution using 15 μL of Lipofectamine LTX Reagent and 150 μL of Opti-MEM Medium. We dilute the plasmid DNA in 150 μL of Opti-MEM Medium and then add 14 μL of PLUS™ Reagent. Nevertheless, the amounts of Lipofectamine LTX Reagent, plasmid DNA and PLUS™ Reagent may need to be optimized if alternative systems are used.

***Optional:*** Before adding the PLUS™ Reagent to the diluted DNA, the diluted DNA can be filtered using a 0.2 μM PES syringe filter. To do this at least four times as much diluted DNA solution in Opti-MEM medium should be prepared to avoid losing the DNA when filtering. 150 μL of the filtered DNA solution can then be transferred to a new Eppendorf before adding the PLUS™ Reagent and performing the rest of the protocol as normal.

### Cell surface labeling experiments

**Timing: 4 days**

This step is performed to probe isoenzyme-dependent glycosylation in the living cell by visualizing labeled cell surface glycoproteins by in-gel fluorescence.

41. Plate stably transfected K-562 cells at a density of 4 × 10^5^ cells/mL in 1.6 mL of growth medium without hygromycin B into a 6-well plate.
42. Feed cells with 50 μM compound **2**, 50 μM Ac_4_ManNAl as a positive control and DMSO vehicle as a negative control.
43. Incubate cells at 37°C for another 20 h.
44. Centrifuge cells for 5 min at 500 × *g* and 4°C.
45. Resuspend cells in 200 μL of ice-cold 2% FBS in 1× PBS and transfer to a V-shaped 96-well plate.
46. Centrifuge for 3 min at 500 × *g* and 4°C.
47. Discard the supernatant.
48. Wash the cells twice with 200 μL of ice-cold 2% FBS in 1× PBS.
49. Resuspend the cells in 35 μL of 2% FBS in 1× PBS.
50. Treat the cells with 35 μL of 2× CuAAC solution I.
51. Incubate the 2× CuAAC reaction mixture for 7 min at 20°C–25°C on an orbital shaker.
52. Quench the CuAAC reaction with 35 μL of quenching solution.
53. Centrifuge for 3 min at 500 × *g* and 4°C.
54. Discard the supernatant.
55. Wash the cells twice with 200 μL of PBS.
56. Resuspend the cells in 100 μL of ice-cold lysis buffer I.
57. Lyse the cells for 20 min at 4°C on an orbital shaker.
58. Centrifuge for 20 min at 1500 × *g* and 4°C.
59. Transfer the supernatant to a new plate.
60. Measure protein concentration by BCA.
  a. We use the Pierce™ BCA Protein Assay Kit according to the manufacturer’s instructions (https://www.thermofisher.com/document-connect/document-connect.html?url=https%3A%2F%2Fassets.thermofisher.com%2FTFS-Assets%2FLSG%2Fmanuals%2FMAN0011430_Pierce_BCA_Protein_Asy_UG.pdf&title=VXNlciBHdWlkZTogUGllcmNlIEJDQSBQcm90ZWluIEFzc2F5IEtpdA==).
61. Treat equal amounts of protein (typically 15 μg) with PNGase F (4 μL of diluted PNGase F solution) and make up to 40 μL with lysis buffer I.
62. Incubate samples for 16–20 h at 37°C.
63. Quench by heating at 95°C for 10 s before placing on ice.
64. Labeling analysis by in-gel fluorescence (troubleshooting 4 and 5):
  a. Add 4× loading buffer to each sample.
    i. The exact amount of loading buffer to be used will depend on the volume of sample to be loaded on the gel.
    ii. Dilute 3 parts of sample with 1 part of 4× loading buffer so that the final concentration of loading buffer in the mixture is 1×.
  b. Run the samples on a 4%–20% gel for SDS-PAGE.
  c. Incubate the gel in fixing solution for 10 min at 20°C–25°C on an orbital shaker.
  d. Wash the gel once with deionized water.
  e. Image the gel on a fluorescence gel imager.
  f. Incubate the gel in SafeBlue Protein Stain for 30 min to assess total protein content.
  g. Discard SafeBlue Protein Stain.
  h. Wash the gel twice with deionized water.
  i. Image the gel on a fluorescence gel imager.
65. Protein expression control by Western blot:
  a. Add 4× loading buffer to each sample.
  b. Boil samples at 95°C for 5 min.
  c. Run the samples on a 4%–20% gel for SDS-PAGE.
  d. Transfer the gel to a nitrocellulose membrane.
    i. We use the Trans-Blot Turbo Transfer System according to the manufacturer’s instructions (https://www.bio-rad.com/webroot/web/pdf/lsr/literature/10016505E.pdf).
    ii. From the optimized protocols integrated in the Trans-Blot Turbo Transfer System we use the MIXED MW protocol (5–150 kDa, 7 min, 2.5 A constant up to 25 V for 2 Mini Gels or 1 Midi Gel / 2.5 A constant up to 25 V for 1 Mini Gel).
  e. Once the transfer is complete, leave the membrane to dry to maximize protein retention.
  f. Wet the membrane with deionized water until fully hydrated.
  g. Incubate the membrane in 5 mL of Revert™ 700 Total Protein Stain for 5 min.
  h. Rinse the membrane twice with 5 mL of Revert™ 700 Wash Solution.
  i. Wash the membrane with deionized water.
  j. Image the membrane on a fluorescence gel imager.
  k. Incubate the membrane in 5 mL of Revert™ Destaining Solution for 5 min.
  l. Wash the membrane with deionized water.
  m. Incubate the membrane in blocking buffer for 1 h at 20°C–25°C.
  n. Perform antibody staining.
  o. Image the membrane on a fluorescence gel imager.

**CRITICAL:** As the CF680 picolyl azide is light-sensitive, once the CuAAC solution is added to the samples these should be covered with aluminum foil. The Revert™ Destaining Solution should not be left for longer than 10 min. Compound **1** has been shown to be accepted by GALE,^1^ an epimerase which catalyzes the interconversion of UDP-GalNAc and UDP-GlcNAc in living cells. This means that the alkyne-containing UDP-GlcNAc analogue product of the epimerization reaction may be incorporated into GlcNAc-containing extracellular glycoproteins, such as N-glycans. PNGase F treatment of the cell lysates is therefore crucial to remove N-glycans prior to analysis. This helps discern between N- and O-glycans and reduces background fluorescence.

***Note:*** The cell lysates can be stored at −20°C for short-term storage or at −80°C for long-term storage. We recommend using K-562 cells to assess whether the approach works. Other cell lines may need optimization in constructs, sugar concentration and feeding time. We also recommend using constructs with both AGX1 and GalNAc-T on the same plasmid. Co-transfection or sequential transfection has led to inferior labeling outcomes in the past. This may be particularly important in the case of cell lines that make elaborated O-glycans, as these likely incorporate the GlcNAc derivative into their O-glycans. Many cancer cell lines have short O-GalNAc glycans and are easier to use in this procedure. Analyzing in-gel fluorescence from glycoproteins labeled with the CF680 fluorophore using a laser-scanning system has given the best results. Other read-outs e.g., using alternative fluorophores, camera-based imagers or blots to visualize the labelled glycoproteins have led to lower signals. We use an Odyssey CLx Imager and Image Studio software for image acquisition and processing.

***Optional:*** An expression vector containing VSV-G-tagged WT/BH GalNAc-T under the control of a Dox-inducible promoter may be used as a control to assess background protein labeling in the absence of the GalNAc-T. GALE-KO cells may be used to prevent the epimerization of the compound **1** to the corresponding UDP-GlcNAc analogue by GALE. PNGase F treatment would not be necessary in this case.

### Sample preparation for MS-proteomics and glycoproteomics

**Timing: 2 weeks**

This step is performed to prepare peptide and glycopeptide fractions from lysates of MCF7 cells transfected with the BH GalNAc-T system for subsequent MS-proteomics and glycoproteomics.

66. Plate stably transfected cells at a density of 2.4 × 10^5^ cells/mL in 25 mL of growth medium without hygromycin B into a 15 cm dish.
67. Incubate cells for 16–20 h at 37°C to allow the cells to adhere to the plate.
68. Feed cells with 10 μM compound **3** or DMSO.
69. Incubate cells at 37°C for another 20 h.
70. Discard media.
71. Wash cells with 5 mL of PBS.
72. Incubate cells for 10 min with 8 mM EDTA in 1× PBS.
73. Detach cells by pipetting using a 1 mL pipette and transfer to a centrifuge tube on ice.
74. Centrifuge cells for 5 min at 500 × *g* and 4°C.
75. Wash cells with 200 μL of PBS.
76. Centrifuge for 3 min at 500 × *g* and 4°C.
77. Discard the supernatant.
78. Repeat wash with PBS.
79. Resuspend cells in 200 μL of ice-cold lysis buffer II.
80. Lyse the cells for 20 min at 4°C on an orbital shaker.
81. Centrifuge for 20 min at 1500 × *g* and 4°C.
82. Transfer supernatant to low-bind tubes.
83. Measure protein concentration by BCA.
  a. We use the Pierce™ BCA Protein Assay Kit according to the manufacturer’s instructions (https://www.thermofisher.com/document-connect/document-connect.html?url=https%3A%2F%2Fassets.thermofisher.com%2FTFS-Assets%2FLSG%2Fmanuals%2FMAN0011430_Pierce_BCA_Protein_Asy_UG.pdf&title=VXNlciBHdWlkZTogUGllcmNlIEJDQSBQcm90ZWluIEFzc2F5IEtpdA==).
84. Dilute equal amounts of protein (300 μg) to 250 μL with PBS.
85. Transfer 300 μL of Sera-Mag SpeedBeads Neutravidin-Coated Magnetic Beads slurry to low-bind tubes.
86. Place the tubes on a magnetic rack and wait until the beads have migrated to the tube wall.
87. Discard the supernatant.
88. Add 200 μL of PBS.
89. Vortex briefly to resuspend the beads.
90. Spin down to ensure that all the solution is at the bottom of the tube.
91. Place the tubes on a magnetic rack and discard the supernatant.
92. Repeat wash with 200 μL of PBS.
93. Add samples to the beads.
94. Vortex briefly to ensure the beads are in solution.
95. Incubate for 2 h at 20°C–25°C under rotation.
  a. This step removes endogenous biotinylated proteins from the samples.
96. Spin down to ensure that all the solution is at the bottom of the tube.
97. Place the tubes on a magnetic rack.
98. Collect the supernatant.
99. Dilute samples to 270 μL with PBS.
100. Treat samples with 30 μL of 10× CuAAC solution II.
101. Incubate samples for 6 h at 20°C–25°C under shaking (400 rpm).
102. Precipitate samples with 3 mL of cold methanol (10-fold excess) for 16–20 h at −80°C.
103. Centrifuge samples for 20 min at 3700 × *g* and 4°C.
104. Discard supernatant.
105. Add 3 mL of cold methanol (10-fold excess).
106. Centrifuge samples for 20 min at 3700 × *g* and 4°C.
107. Discard supernatant.
108. Repeat wash with 3 mL of cold methanol.
109. Discard supernatant.
110. Place the tubes upside down on tissue paper and air-dry the pellets to completely remove the methanol.
111. Resuspend pellets with 250 μL of 0.1% (w/v) RapiGest in PBS.
112. Sonicate samples in a water bath for 25 min.
113. Centrifuge samples for 5 min at 3700 × *g.*
114. Collect supernatant.
115. Resuspend pellets with 250 μL of 6 M urea in PBS.
116. Sonicate samples in a water bath for 25 min.
117. Centrifuge samples for 5 min at 3700 × *g.*
118. Collect supernatant.
119. Resuspend pellets with 250 μL of PBS.
120. Sonicate samples in a water bath for 25 min.
121. Centrifuge samples for 5 min at 3700 × *g.*
122. Collect supernatant.
123. Combine RapiGest, urea and PBS supernatants.
124. Transfer 350 μL of Lys-dimethylated Sera-Mag SpeedBeads Neutravidin-Coated Magnetic Beads slurry to low-bind tubes.
125. Place the beads on a magnetic rack and wait until the beads have migrated to the tube wall.
126. Discard the supernatant.
127. Add 200 μL of PBS.
128. Vortex briefly to resuspend the beads.
129. Spin down to ensure that all the solution is at the bottom of the tube.
130. Place the tubes on a magnetic rack and discard the supernatant.
131. Repeat wash with 200 μL of PBS.
132. Add the combined RapiGest, urea and PBS supernatants to the beads.
133. Vortex briefly to ensure the beads are in solution.
134. Incubate for 2 h at 20°C–25°C under rotation.
135. Wash beads three times with 350 μL of 1% (w/v) RapiGest in PBS.
136. Wash beads six times with 350 μL of 6 M urea in PBS.
137. Wash beads six times with 350 μL of AmBic.
138. Wash beads four times with 100 μL of 40% (v/v) LC/MS-grade acetonitrile in LC/MS-grade water.
139. Resuspend beads in 100 μL of AmBic containing 10 mM DTT to reduce disulfide bonds.
140. Incubate beads for 15 min at 50°C under shaking at 400 rpm.
141. Wash beads twice with 350 μL of AmBic.
142. Add 100 μL of 20 mM iodoacetamide in AmBic to alkylate the reduced disulfide bonds.
143. Incubate beads in the dark for 30 min at 20°C–25°C.
144. Add 100 μL of AmBic containing 10 mM DTT to neutralize iodoacetamide.
145. Discard supernatant.
146. Wash beads three times with 350 μL of AmBic.
147. Resuspend beads with 100 μL of AmBic.
148. Add 300 ng of Lys-C (i.e., 3 μL of the 0.1 μg/μL stock) to the beads.
149. Incubate samples for 16–20 h at 37°C under shaking at 400 rpm.
150. Prepare samples for proteomics:
  a. Transfer supernatant to a new tube.
  b. Add 200 ng of trypsin (i.e., 2 μL of the 0.1 μg/μL stock) to the samples.
  c. Incubate samples for 8 h at 37°C under shaking at 400 rpm.
  d. Vacuum-dry samples by SpeedVac.
151. Prepare samples for glycoproteomics following on-bead Lys-C digest:
  a. Add 150 μL of 1% (v/v) formic acid in LC/MS-grade water to the beads.
    i. This will cleave the acid-cleavable biotin probe and release the glycopeptides bound to the beads.
  b. Incubate samples for 30 min at 20°C–25°C on a rotator.
  c. Collect supernatant.
  d. Repeat acid treatment.
  e. Wash beads with 150 μL of LC/MS-grade acetonitrile.
  f. Combine wash and collected supernatants.
  g. Vacuum-dry samples by SpeedVac.
  h. Reconstitute samples in 100 μL of AmBic.
  i. Add 200 ng of trypsin (i.e., 2 μL of the 0.1 μg/μL stock) to the samples.
  j. Incubate samples for 8 h at 37°C under shaking at 400 rpm.
  k. Vacuum-dry samples by SpeedVac.
152. Desalt peptides and glycopeptides using UltraMicroSpin™ columns:
  a. Place the UltraMicroSpin™ column in a 1.5 mL centrifuge tube.
  b. Condition the column:
    i. Add 100 μL of conditioning solvent to the column.
    ii. Use a 1 mL syringe attached to an applicator to push the liquid through the column.
    iii. Discard flow-through.
    iv. Repeat conditioning step.
  c. Load sample:
    i. Reconstitute sample in 100 μL of loading buffer.
    ii. Load sample on the column.
    iii. Use a 1 mL syringe attached to an applicator to push the liquid through the column.
    iv. Discard flow-through.
  d. Wash column:
    i. Add 50 μL of wash buffer to the column to wash any traces of salts.
    ii. Use a 1 mL syringe attached to an applicator to push the liquid through the column.
    iii. Discard flow-through.
  e. Elute sample:
    i. Place the UltraMicroSpin™ column in a clean 1.5 mL centrifuge tube.
    ii. Add 50 μL of elution buffer to the column.
    iii. Use a 1 mL syringe attached to an applicator to push the liquid through the column.
    iv. Collect flow-through.
  f. Vacuum-dry the eluted sample to remove any traces of organic solvent.
    i. Store eluted sample at −80°C.

**CRITICAL:** From the protein precipitation in methanol step onwards all reagents used should be LC-MS grade. To characterize O-GalNAc glycans specifically, the lysates should be treated with PNGase F prior to the click reaction or GALE KO cells should be used for the experiment. We recommend using Protein LoBind® Tubes throughout the procedure to minimize sample loss due to protein-surface binding.

**Pause point:** The whole cell lysates can be stored at –20°C prior to the click reaction. The clicked lysates can also be stored at –20°C prior to the protein precipitation. Combined supernatants after protein precipitation and resuspension can be stored at –20°C prior to enrichment. The dried pellets before sample-desalting can be stored at –80°C. The dried pellets after sample-desalting can be stored at −80°C.

***Note:*** The protein expression levels, sugar feeding conditions and click reaction efficiency may vary between cell lines so the experimental conditions might need to be optimized. The cells should be 30%–60% confluent at the time of sugar feeding. We recommend detaching the cells by pipetting using a 1 mL pipette since we have seen that scraping can lead to a lower cell viability.

***Optional:*** When desalting the peptides, the flow-through may be collected after loading the sample and washing the column in case not all the sample is retained in the column. If the secretome is to be analyzed instead of whole cell lysates the procedure is the same with the exception that serum-free medium should be used and two 15 cm dishes should be prepared per sample instead of one. After sugar feeding and incubation for 16–20 h, the secretome can be collected and harvested to remove cellular debris. The samples can then be concentrated to 200 μL using Amicon Ultra-15 Centrifugal Filters (3 kDa MWCO) and the medium exchanged with PBS. Even though we haven’t done this ourselves, if one would like to estimate the amount of protein bound to the neutravidin beads during the enrichment step we believe that the AVIDITY assay^34^ may be applicable for this purpose. The beads would have to be first incubated with HABA, the supernatant collected and the absorbance of free HABA at 350 nm measured. The supernatant would then be returned to the beads and the samples added. The supernatant would be collected and the absorbance at 350 nm measured again. Since the biotinylated glycoproteins in the samples will displace the HABA bound to the beads, measuring the change in absorbance due to free HABA in the supernatant before and after adding the samples to the beads could be used to infer the amount of proteins bound to the beads.

### Mass spectrometry data acquisition

**Timing: 1 day**

This step is performed to acquire the raw MS data from the peptide and glycopeptide fractions from lysates of cells transfected with the BH GalNAc-T system.

153. Prepare Buffer A: 0.1% (v/v) formic acid in LC/MS-grade water.
154. Prepare Buffer B: 0.1% (v/v) formic acid in LC/MS-grade acetonitrile.
155. Reconstitute your dried samples in 16 μL of Buffer A.
156. Sonicate samples in a water bath for 15 min.
157. Vortex the samples briefly.
158. Centrifuge samples for 5 min at 18 000 × *g.*
159. Transfer the supernatant to a glass screw neck vial.
160. Load samples via autosampler and inject using a flow rate of 0.3 μL/min onto a 75 μm × 150 mm EASY-Spray column containing 2 μm C18 beads.
161. Hold columns at 40°C using a column heater in the EASY-Spray ionization source.
162. Chromatographically separate the sample using a 75 min gradient and a 105 min instrument method.
163. For proteomic analyses, set up the instrument to acquire data in a dependent fashion using only higher-energy collisional dissociation (HCD).
  a. Instrument method details are as follows:
    i. Full mass spectra should have an MS1 precursor mass resolution set to 60,000 at full width at half maximum (FWHM) 400 m/z, a mass range of 350–1,500 m/z, and sample charge states 2–6.
    ii. Generate HCD data with the Set the precursor automated gain control (AGC) settings to 3e5 ions and set the isolation window for HCD to 1.6 Da and the collision energy to 30.
    iii. Enable dynamic exclusion with a repeat count of 3, repeat duration of 10 s, and an exclusion duration of 10 s.
    iv. MS2 spectra should be generated using an Orbitrap at top speed for 3 s.
164. For glycoproteomic analyses, set up the instrument to acquire data in a dependent fashion using HCD product dependent electron transfer dissociation (HCD-pd-ETD).
  a. Details on the instrument method are as follows:
    i. Full mass spectra should have an MS1 precursor mass resolution set to 60,000 at FWHM 400 m/z, a mass range of 350–1,500 m/z, and sample charge states 2–6.
    ii. Generate HCD data with the Set the precursor AGC settings to 3e5 ions and set the isolation window for HCD to 1.6 Da and the collision energy to 30.
    iii. Enable dynamic exclusion with a repeat count of 3, repeat duration of 10 s, and an exclusion duration of 10 s.
    iv. MS2 spectra should be generated at top speed for 3 s.
    v. To enable HCD-pd-ETD, select “Targeted Mass Trigger” and perform ETD if: (a) the precursor mass is between 300 and 1000 m/z and (b) in the HCD spectrum, 2 of 10 glyco-fingerprint ions (126.055, 138.055, 144.07, 168.065, 186.076, 204.086, 274.092, 292.103; 491.2241, 330.1554) are present at +/- 20 ppm and greater than 5% relative intensity.
    vi. Set ETD parameters as follows: calibrated charge-dependent ETD times, 2e5 reagent target, and precursor AGC target 1e4.
    vii. Read out fragment ions in the ion trap using centroid mode.
165. Set up the gradient profile as follows: (minute:%B): 0:2, 5:2, 80:35, 85:95, 90:95, 92:2, 105:2.

***Note:*** Due to the labile nature of glycan modifications, we recommend running glycopeptide samples on an Orbitrap Eclipse with ETD (Thermo Fisher) coupled to an UltiMate 3000 RSLCnano. For the peptide samples we perform three 5 μL injections per sample to have technical replicates, whilst for the glycopeptide samples we perform one 15 μL injection instead. Additionally, these settings can vary depending on the HPLC and mass spectrometer being used and should be optimized for different systems.

### Mass spectrometry data analysis of peptides using MaxQuant and Perseus

**Timing: 2 days**

This step is performed to analyze the raw MS data from the peptide fraction from lysates of cells transfected with the BH GalNAc-T system by label-free quantitative analysis to dissect the protein substrates of the GalNAc-T of interest.

166. Perform a database search using MaxQuant (Methods video S1).
  a. Load raw mass spectrometry files.
  b. Select Set experiment to name each sample.
    i. If you want to treat your technical replicates as a single experiment, they should have the same name in the Experiment column.
  c. In the Group-specific parameters section:
    i. In Type select Standard and Multiplicity 1.
    ii. In Modifications include methionine oxidation and N-terminal acetylation as variable modifications and cysteine carbamidomethylation as a fixed modification with a total common max of 5.
    iii. In Instrument modify the default parameters based on the instrument used.
    iv. In Digestion mode select Specific and in Enzyme select Trypsin/P. Allow two missed cleavages.
    v. In Label free quantification select None as no specific labeling /quantification strategy was applied to the samples.
  d. In the Global parameters section:
    i. In Sequences add the *Homo sapiens* FASTA file downloaded from UniProt.
    ii. If the Identifier rule column is not automatically filled once the FASTA file is added, click on the Identifier rule button and select the UniProt identifier.
    iii. Click on the Test button to test the parse rules applied to retrieve the identifiers.
    iv. Tick to include contaminants.
    v. In Label free quantification tick iBAQ.
  e. Select the number of processors based on the instrument used.
  f. Press Start to initiate the search.
167. Visualize the results from the database search using Perseus (Methods video S2).
  a. Upload the proteinGroups.txt file.
    i. In Main select the iBAQ for each sample.
    ii. In Categorical select Taxonomy IDs and Peptide IDs.
    iii. Leave the other default parameters untouched.
    iv. Select OK.
  b. Filter the rows of the matrix produced based on the Reverse categorical column.
    i. In column select Reverse.
    ii. In mode select Remove matching rows.
    iii. In filter mode select Reduce matrix.
    iv. Select OK.
  c. Filter the rows of the matrix produced based on the Potential contaminant categorical column.
    i. In column select Potential contaminant.
    ii. In mode select Remove matching rows.
    iii. In filter mode select Reduce matrix.
    iv. Select OK.
  d. In the Annotate rows function select Categorical annotation rows.
    i. In Row name type Technical replicates.
    ii. For each sample make sure the replicates have the same name for the iBAQ columns (e.g., Exp1, Exp2, ...).
    iii. Select OK.
  e. In the Annotate columns function select Add annotation.
    i. In Source select the .txt file containing the annotations for *Homo sapiens* derived from Uniprot and downloaded from http://annotations.perseus-framework.org.
    ii. In UniProt column select Protein IDs.
    iii. In Annotations to be added select Corum, Keywords, Taxonomy and UniProt names.
    iv. Select OK.
  f. In the Normalization function select Subtract.
    i. In Matrix access select Columns.
    ii. In Subtract what select Median.
    iii. Select OK.
  g. Use the Transform function on the matrix produced to transform the data to a logarithmic base.
    i. In Transformation select log2(x).
    ii. In Columns select the iBAQ columns.
    iii. Select OK.
  h. Use the Replace missing values from normal distribution function to make sure that all the datapoints have a numerical value.
    i. Leave default parameters for Width, Down shift and Mode untouched.
    ii. In Columns select the iBAQ columns.
    iii. Select OK.
  i. Use the Two sample test function to statistically analyze the data.
    i. In Grouping select Technical replicates.
    ii. In First group (right) and Second group (left) select the two samples to be analyzed.
    iii. In Test select Welch’s T-test.
    iv. In Use for truncation select p-value.
    v. Select OK.
  j. Use the Scatter plot function to visualize the data produced.
    i. On the x-axis select Welch’s T-test difference.
    ii. On the y-axis select -Log Welch’s T-test p-value.
    iii. Hits will be considered if they have a -log Welch T-test p-value greater than 1.3 and a Welch T-test difference greater than 3 (i.e., eightfold enrichment).

***Note:*** If cells from species other than human are used, the FASTA file from the corresponding species should be added instead when performing the database search with MaxQuant e.g., if murine cells are used the Mus musculus FASTA file should be uploaded instead. The avidin FASTA file can also be downloaded from Uniprot and uploaded to MaxQuant to identify any contaminants in the samples from the neutravidin beads due to the enrichment process.

Methods video S1. Representative video of how to perform a database search using MaxQuant for MS analysis of peptides, related to step 166

Methods video S2. Representative video of how to visualize the results from the MaxQuant database search using Perseus for MS analysis of peptides, related to step 167

### Mass spectrometry data analysis of glycopeptides using Byonic

**Timing: 1 week**

This step is performed to analyze the raw MS data from the glycopeptide fraction from lysates of cells transfected with the BH GalNAc-T system to identify GalNAc-T-specific glycosylation sites.

168. Search your raw files with 10 ppm mass tolerance for precursor mass ions, with 20 ppm and 0.2 Da fragment mass tolerances for HCD and ETD fragmentation, respectively.
169. Allow up to two missed cleavages per peptide and semi-specific, C-terminal tryptic digestion (R,K Cleavage sites).
  a. Use a 1% false discovery rate using standard reverse-decoy techniques.
170. Methionine oxidation (common 1) and asparagine deamidation (common 1) should be set as variable modifications with a total common max of 2, rare max of 1.
  a. Carbamidomethyl should be set as a fixed modification.
171. Under the “Advanced” tab in the Byonic interface, select “Create focused database” and start the search.
  a. This will create a FASTA file containing only the proteins that Byonic finds in the sample, which can then be used to reduce search times with glycans added.
172. Edit the existing Byonic parameter file by selecting the focused database FASTA file.
173. Under the “Glycans” tab, add the following modifications (Table 2):

**Table 2:** Glycan modifications to be added in the “Glycans” tab in Byonic

**Figure 7:**
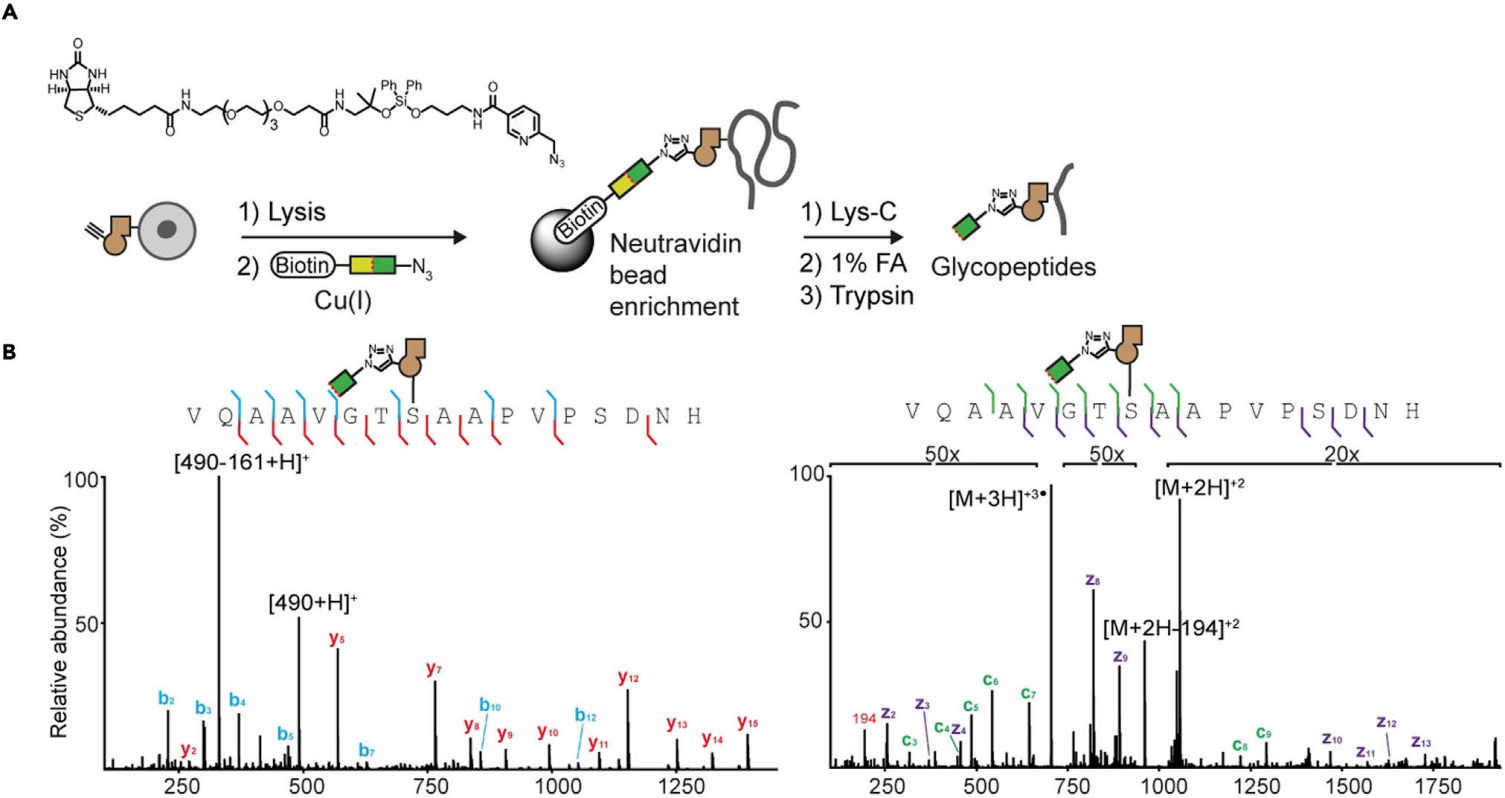
GalNAc-T protein substrate and glycosylation site identification by MS-glycoproteomics (A) Schematic of MS-glycoproteomics workflow. Following cell lysis, the labelled glycoproteins are derivatised with biotin-DADPS-picolyl azide (which contains an acid-labile diphenyldisiloxane moiety) and enriched for using neutravidin beads. On-bead Lys-C digestion releases non-glycosylated peptides. Labelled glycopepetides are eluted from the beads by acid treatment, digested with trypsin and analysed by MS-glycoproteomics. Figure adapted from Schumann et al.^1^ (Figure 5D). (B) Exemplary mass spectra from a glycopeptide of ApoE and confirmation of Ser308 as the glycosylation site after modification by BH GalNAc-T2. HCD (mainly glycan fragmentation, left) and ETD (mainly peptide fragmentation, right) spectra are shown, and ions are annotated. y and b ions in HCD spectra were devoid of glycan. Figure reprinted from Molecular Cell, Vol 78/5, B. Schumann et al., Bump-and-Hole Engineering Identifies Specific Substrates of Glycosyltransferases in Living Cells, 824–834.e15, Copyright (2020) (Figure S5D).^1^

***Note:*** For a more detailed explanation on mass spectrometry analysis using Byonic please refer to Malaker et al.^36^ We use Byonic to analyse the data from the glycopeptide fraction but alternative software can be used instead.

Glycan modifications
% Custom modification text below HexNAc(1)Hex(1)NeuAc(2) 287.1389 @ OGlycan | common2 HexNAc(1) 287.1389 @ OGlycan | common2 HexNAc(1)Hex(1) 287.1389 @ OGlycan | common2 HexNAc(1)NeuAc(1) 287.1389 @ OGlycan | common2 HexNAc(1)Hex(1)NeuAc(1) 287.1389 @ OGlycan | common2 HexNAc(2)Hex(1)NeuAc(1) 287.1389 @ OGlycan | common2 HexNAc(1)NeuGc(1) 287.1389 @ OGlycan | common2 HexNAc(1)Hex(1)NeuGc(1) 287.1389 @ OGlycan | common2 HexNAc(1)Hex(1)NeuGc(2) 287.1389 @ OGlycan | common2 HexNAc(1)Hex(1)NeuAc(1)NeuGc(1) 287.1389 @ OGlycan | common2 HexNAc(2)Hex(1)NeuGc(1) 287.1389 @ OGlycan | common2 HexNAc(3)Hex(2) 287.1389 @ OGlycan | common2 HexNAc(3)Hex(2)NeuAc(1) 287.1389 @ OGlycan | common2 HexNAc(3)Hex(2)NeuGc(1) 287.1389 @ OGlycan | common2 HexNAc(3)Hex(2)Fuc(1)NeuAc(1) 287.1389 @ OGlycan | common2 HexNAc(3)Hex(2)Fuc(1)NeuGc(1) 287.1389 @ OGlycan | common2 HexNAc(3)Hex(3) 287.1389 @ OGlycan | common2 HexNAc(3)Hex(3)Fuc(1) 287.1389 @ OGlycan | common2 HexNAc(3)Hex(3)NeuAc(1) 287.1389 @ OGlycan | common2 HexNAc(3)Hex(3)NeuGc(1) 287.1389 @ OGlycan | common2 HexNAc(2)Hex(2)Fuc(2) 287.1389 @ OGlycan | common2 HexNAc(3)Hex(3)NeuAc(1)NeuGc(1) 287.1389 @ OGlycan | common2
174. Search the data again with these parameters.
175. After the search finishes, open the excel file of the output and sort by “Glycans” (troubleshooting 6).
  a. Move the rows with any glycans annotated to a new sheet and sort by |Log Prob|.
  b. Remove anything with a Log Prob value of < 3.
  c. Similarly, sort the file by Score and remove anything with a value of < 100.
176. Sort the excel file by “Protein Rank” and open the Byonic Viewer.
  a. Manually examine the HCD spectra of any remaining glycopeptides in the excel file.
  b. Each HCD spectrum with a GalN6yne modified peptide should contain an oxonium ion at 491 and another at 330.
  c. Additionally, the peptide should have at least 50% of the naked (i.e., non-glycosylated) b/y ions annotated.
  d. If the peptide meets these criteria, then move on to the next step, otherwise the peptide is not correctly annotated and should not be counted as a modified glycopeptide.
177. Extract the associated chromatograph in Qual Browser along with the associated ETD MS2 spectra.
178. Average the MS2 spectra generated and *de novo* sequence the glycopeptide to identify which site is modified (Figure 7).
  a. A detailed tutorial for manual interpretation of ETD spectra is available here.^35^

## Expected outcomes

**Figure 8:**
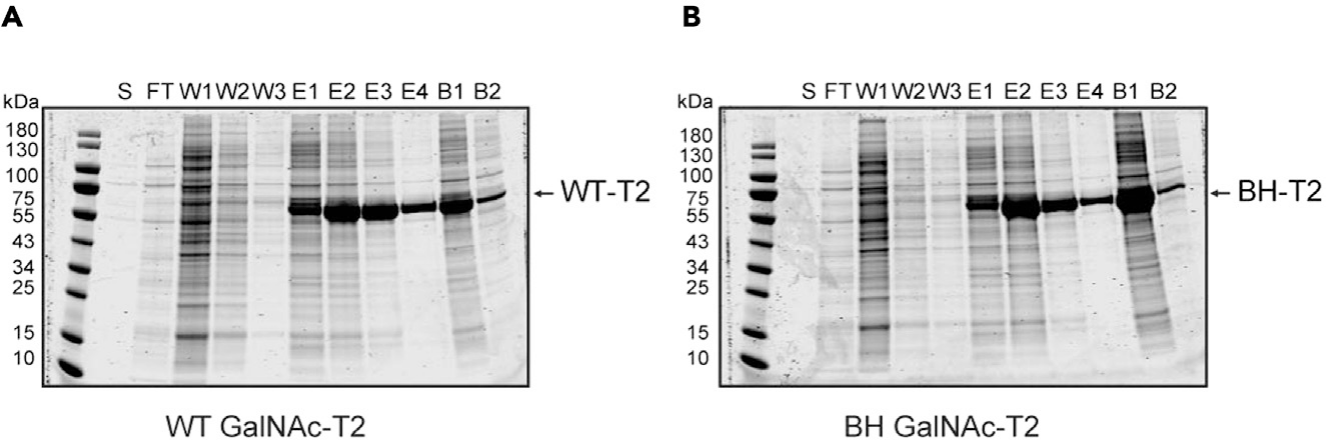
SDS-PAGE gels of His-tagged truncated WT and BH GalNAc-Ts following secreted protein expression and Ni-NTA purification (A and B) Representative SDS-PAGE gels of His-tagged truncated (A) WT and (B) BH GalNAc-T2 following secreted protein expression and Ni-NTA purification. S = supernatant, FT = flow-through of the unbound fraction, W = wash, E = elution (with 100 mM, 2 × 200 mM and 300 mM imidazole for E1, E2, E3 and E4 respectively), B1 = beads before elution, B2 = beads after elution. The predicted molecular weight of truncated GalNAc-T2 is 58 kDa (arrows pointing). The E3 and E4 fractions were pooled together and the protein concentration determined by Nanodrop (WT GalNAc-T2 = 0.37 mg/mL and BH GalNAc-T2 = 0.276 mg/mL).

After secreted protein expression and Ni-NTA purification of WT and BH GalNAc-T, SDS-PAGE gels can be run to evaluate the success of the expression and purification of the enzymes (Figure 8). A band at the expected molecular weight of the protein should be seen in the fractions, with most of the purified protein observed in the eluted fractions. Ideally the eluted fraction chosen to be used in downstream applications should contain as few impurities as possible, as these could interfere with the activity of the enzyme.

When evaluating the activity of the purified enzymes by *in vitro* glycosylation experiments, the WT enzyme should show activity with its native substrate (UDP-GalNAc) exclusively, whereas the BH GalNAc-T should only show activity with the corresponding “bumped” analogue (compound **1**).

When performing the *in vitro* glycosylation experiments with a small panel of synthetic peptides, the glycosylation profile of the BH GalNAc-T should match that of the corresponding WT GalNAc-T in terms of peptide substrate specificity and glycopeptide formation.

**Figure 9:**
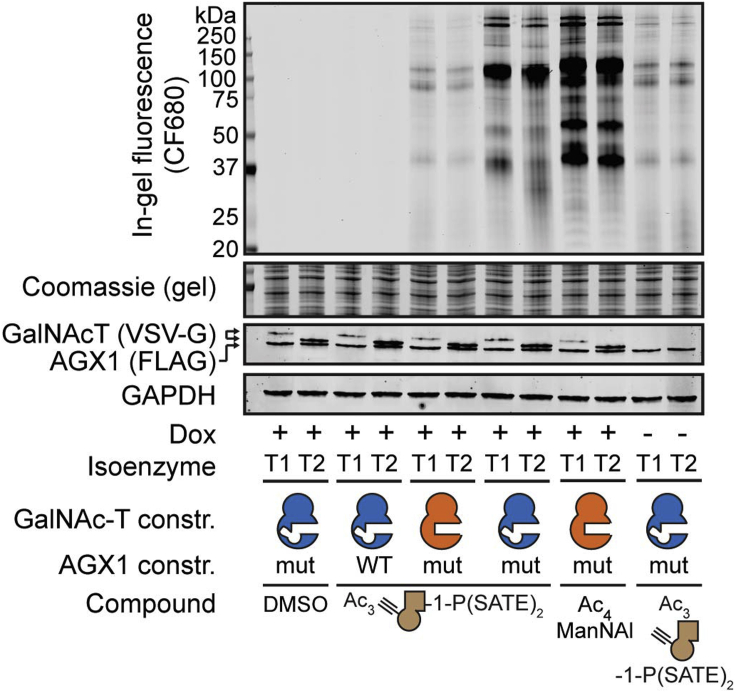
Representative in-gel fluorescence analysis and protein expression control by Western blot of PNGase F-treated lysates from metabolically labeled K-562 cells transfected with AGX1 (WT or mut) and either GalNAc-T1 or T2 (WT or BH mutant) constructs In this experiment GalNAc-T expression was under the control of a Dox-inducible promoter. Figure reprinted from Molecular Cell, Vol 78/5, B. Schumann et al., Bump-and-Hole Engineering Identifies Specific Substrates of Glycosyltransferases in Living Cells, 824–834.e15, Copyright (2020) (Figure S4F).^1^

If the cell surface labeling experiments have been successful, labeling should be observed in the cells fed with Ac_4_ManNAl whilst no labeling should be seen in cells fed with DMSO. In cells fed with the compound **2**, the strongest labeling should be observed when mut-AGX1 and BH GalNAc-T are present in the cell (Figure 9). Any background labeling due to GlcNAc-containing glycoproteins should be removed upon treatment with PNGase F, in particular in the cells fed with Ac_4_ManNAl as the sugar is a N-acetylneuraminic acid (Neu5Ac) precursor and Neu5Ac is known to typically cap N-glycans. Different GalNAc-Ts should produce slightly different band patterns, reflecting the different protein substrate specificities of the different isoenzymes.

## Quantification and statistical analysis

### *In vitro* glycosylation experiments

At least three independent replicates should be performed for each experiment. Each independent replicate should consist of two technical replicates for each UDP-sugar/peptide used.

An average value of glycopeptide formation should be calculated using the two technical replicates for each UDP-sugar/peptide used. The final values should be reported as the mean glycopeptide formation across the three independent replicates ± standard deviation.

### Michaelis-Menten kinetics

For the *in vitro* glycosylation experiments using different enzyme concentrations:

At least three independent replicates should be performed for each experiment. Each independent replicate should consist of two technical replicates for each enzyme concentration used.

An average value of glycopeptide formation should be calculated using the two technical replicates for each enzyme concentration used. The final values should be reported as the mean glycopeptide formation across the three independent replicates ± standard deviation.

For the *in vitro* glycosylation experiments using different UDP-sugar concentrations:

At least three independent replicates should be performed for each experiment. Each independent replicate should consist of two technical replicates for each UDP-sugar concentration used.

An average value of glycopeptide formation should be calculated using the two technical replicates for each UDP-sugar concentration used. This average glycopeptide formation should be divided by the duration of the reaction (5400 s) to calculate the initial rate of reaction at each substrate concentration. A mean value of initial rate of reaction should then be calculated for each UDP-sugar concentration using the results of the three independent replicates.

The mean initial rate of reaction should then be plotted against UDP-sugar concentration, with error bars representing the standard deviation. The kinetics parameters can then be calculated as explained above.

## Limitations

The BH GalNAc-T will be in competition for protein substrates and glycosylation sites with the corresponding WT GalNAc-T. This may lead to misleading glycoproteomic analyses. This limitation might be overcome by knocking out the endogenous gene encoding the GalNAc-T of interest by CRISPR-Cas9 followed by stable transfection of the BH GalNAc-T. Alternatively, if compensatory mechanisms take place between the knockout of the endogenous GalNAc-T and the transfection of the BH GalNAc-T, homology-directed repair (HDR) editing can be performed instead to mutate the native allele to the corresponding BH mutant.

The sugar modification may have functional consequences such as affecting antibody/ glycan-binding protein recognition, protein-protein interactions, protein stability and trafficking. This means that this system may have limited use in determining the biological function of glycosylation on its protein substrates.

Similarly, if there are significant differences between the kinetic parameters of the WT GalNAc-T/UDP-GalNAc and BH GalNAc-T/compound **1** pairs, this may have biological consequences in the cell. Nevertheless, the BH enzyme-substrate pairs investigated to date have shown comparable kinetic parameters to their native counterparts.

When the cells are transfected with pSBbi plasmids, the BH GalNAc-T will be overexpressed compared to the corresponding endogenous enzyme. The original publication^1^ demonstrated that BH GalNAc-Ts do not introduce new glycosylation sites in their protein substrates so no false-positive hits should be seen even if the BH GalNAc-T is overexpressed. Nevertheless, an ideal discovery tool would assess native glycosylation levels. This limitation can be overcome by using Dox-inducible promoters to fine-tune the expression levels of the BH GalNAc-T.

On a similar note, we have seen that overexpression of GalNAc-Ts results in reduced sialylation of N- and O-glycans, suggesting an alteration in the cellular glycome.^3^ This observation shouldn’t impair the overall aim of identifying the protein substrates of a specific GalNAc-T but it means that this approach doesn’t fully replicate the native biological system.

While O-GalNAc glycosylation is found in many types of glycoproteins, it is abundantly present in mucins. However, mucins are challenging to study by MS due to their size, their protease resistance, their dense glycosylation and poor ionizability.^37^ Consequently, this can mean that even if we can successfully label the protein substrates of our GalNAc-T of interest with the modified sugar, we still may not be able to detect and characterize these glycoproteins by glycoproteomics. Treatment of the cell lysates with the mucin-selective protease StcE can facilitate this process by breaking the mucins down into smaller fragments which are more amenable to study by MS.^37^ Recent advances in mucin-selective enrichment strategies and in MS fragmentation methods can also aid in mucin glycoproteomics.^38^

## Troubleshooting

### Problem 1

Problem with amplification of the PCR products during the PCR reaction (step 1).

### Potential solution

The PCR conditions may require optimization, such as performing a gradient PCR to find the most appropriate annealing temperature, increasing the denaturation/extension time and/or increasing the number of cycles (although increasing the number of cycles over 35 may be counterproductive as the dNTPs get depleted and this may result in premature stops and truncated products). Alternatively, a small amount of DMSO (e.g., 1%–4%) can be added to the PCR reaction mixture to help lower the melting temperature of the DNA template – this can be particularly helpful for DNA templates with a high GC content.

### Problem 2

Failure to obtain colonies of the transformed bacteria following the PCR reaction and KLD treatment (step 1).

### Potential solution

The PCR/KLD products may be purified and concentrated prior to the transformation step. We use the NucleoSpin® Gel and PCR Clean-up Kit according to the manufacturer’s instructions for this purpose (https://www.takarabio.com/documents/User%20Manual/NucleoSpin%20Gel%20and%20PCR%20Clean/NucleoSpin%20Gel%20and%20PCR%20Clean-up%20User%20Manual_Rev_04.pdf). The KLD reaction time may also be extended up to 30 min at 37°C to improve the efficiency of the reaction.

### Problem 3

No expression of recombinant WT and BH GalNAc-Ts (step 8).

### Potential solution

The protein expression and purification strategy may require optimization, such as using alternative constructs, using different tags/protein purification strategies and using different expression systems.

### Problem 4

No in-gel fluorescence signal observed in the cell labeling experiments (step 64).

### Potential solution

The efficacy of the CuAAC reaction may vary depending on the source of the reagents, therefore the concentration of the CuAAC reaction components and the duration of the reaction may need to be optimized.

### Problem 5

Labeling seen with WT GalNAc-T in the cell labeling experiments (even following PNGase F treatment of the cell lysates) (step 64).

### Potential solution

This may occur due to saturation of the labeling signal when a high sugar concentration is used. Reducing the sugar concentration, for example to 1 μM, should result in labeling only when BH GalNAc-T and mut-AGX1 are present in the cell. Any labeling signal still observed after reducing the sugar concentration may be due to lingering N-glycans as a result of reduced N-glycan cleavage by PNGase F after the click reaction. If this occurs GALE KO cells should be used instead to prevent the epimerization of compound **1** into the corresponding UDP-GlcNAc analogue and subsequent incorporation into GlcNAc-containing glycoproteins.

### Problem 6

No hits obtained after performing the glycoproteomics analysis on the glycopeptide fraction (step 175).

### Potential solution

The sample preparation may require optimization, such as using different sample clean up techniques after the CuAAC reaction, using endoglycosidases and alternative/additional proteases to Lys-C and trypsin and modifying the MS method. Nevertheless, the peptide fraction should be indicative of whether the GalNAc-T of interest has specific peptide substrates.

## Resource availability

### Lead contact

Further information and requests for renewable resources and reagents should be directed to and will be fulfilled by the lead contact, Benjamin Schumann, b.schumann@imperial.ac.uk. For non-renewable resources such as synthetic compounds, we will fulfill requests to the best of our abilities.

### Materials availability

HepG2-T1-KO, HepG2-T2-KO cells and all cell lines derived from these are subject to an MTA with the University of Copenhagen, and Katrine T. Schjoldager (schjoldager@sund.ku.dk) or Hans H. Wandall (hhw@sund.ku.dk) should be contacted. All other cell lines generated in this study are available through the lead author. Expression plasmids for soluble versions of GalNAc-Ts have been made by Kelley Moremen (moremen@uga.edu) and are available through DNASU. All plasmids generated in this study are available through the lead author; of these, plasmids from addgene are protected by an UBMTA and can be obtained for non-commercial use under an equal UBMTA. Newly synthesized chemicals will be shared to the best of our abilities, and synthetic procedures are in the Supporting Information of the original publication.^1^

## Data Availability

Crystal structures are available in the protein databank (PDB: 6E7I and PDB: 6NQT). The mass spectrometry proteomics and glycoproteomics data have been deposited to the ProteomeXchange Consortium via the PRIDE^39^^,^^40^ partner repository with the dataset identifiers PRIDE: PXD017989 and PRIDE: PXD018048.
